# Triose phosphate utilization in leaves is modulated by whole-plant sink–source ratios and nitrogen budgets in rice

**DOI:** 10.1093/jxb/erad329

**Published:** 2023-08-29

**Authors:** Zhenxiang Zhou, Zichang Zhang, Peter E L van der Putten, Denis Fabre, Michael Dingkuhn, Paul C Struik, Xinyou Yin

**Affiliations:** Centre for Crop Systems Analysis, Department of Plant Sciences, Wageningen University & Research, PO Box 430, 6700 AK Wageningen, The Netherlands; Centre for Crop Systems Analysis, Department of Plant Sciences, Wageningen University & Research, PO Box 430, 6700 AK Wageningen, The Netherlands; Institute of Plant Protection, Jiangsu Academy of Agricultural Sciences, Nanjing, Jiangsu, China; Centre for Crop Systems Analysis, Department of Plant Sciences, Wageningen University & Research, PO Box 430, 6700 AK Wageningen, The Netherlands; CIRAD, UMR AGAP Institut, F-34398 Montpellier, France; UMR AGAP Institut, Univ Montpellier, CIRAD, INRAE, Institut Agro, Montpellier, France; CIRAD, UMR AGAP Institut, F-34398 Montpellier, France; UMR AGAP Institut, Univ Montpellier, CIRAD, INRAE, Institut Agro, Montpellier, France; Centre for Crop Systems Analysis, Department of Plant Sciences, Wageningen University & Research, PO Box 430, 6700 AK Wageningen, The Netherlands; Centre for Crop Systems Analysis, Department of Plant Sciences, Wageningen University & Research, PO Box 430, 6700 AK Wageningen, The Netherlands; Universidade Nova de Lisboa, Portugal

**Keywords:** Adaxial versus abaxial measurement, *Oryza sativa*, panicle pruning, triose phosphate utilization, photorespiration-associated nitrogen assimilation, sink limitation, yellower-leaf modification

## Abstract

Triose phosphate utilization (TPU) is a biochemical process indicating carbon sink–source (im)balance within leaves. When TPU limits leaf photosynthesis, photorespiration-associated amino acid exports probably provide an additional carbon outlet and increase leaf CO_2_ uptake. However, whether TPU is modulated by whole-plant sink–source relations and nitrogen (N) budgets remains unclear. We address this question by model analyses of gas-exchange data measured on leaves at three growth stages of rice plants grown at two N levels. Sink–source ratio was manipulated by panicle pruning, by using yellower-leaf variant genotypes, and by measuring photosynthesis on adaxial and abaxial leaf sides. Across all these treatments, higher leaf N content resulted in the occurrence of TPU limitation at lower intercellular CO_2_ concentrations. Photorespiration-associated amino acid export was greater in high-N leaves, but was smaller in yellower-leaf genotypes, panicle-pruned plants, and for abaxial measurement. The feedback inhibition of panicle pruning on rates of TPU was not always observed, presumably because panicle pruning blocked N remobilization from leaves to grains and the increased leaf N content masked feedback inhibition. The leaf-level TPU limitation was thus modulated by whole-plant sink–source relations and N budgets during rice grain filling, suggesting a close link between within-leaf and whole-plant sink limitations.

## Teaser

While triose phosphate utilization limitation is a biochemical process indicating the carbon sink–source (im)balance within the leaf, this process is modulated by whole-plant source–sink ratios and nitrogen budgets in rice.

## Introduction

CO_2_ response curves of leaf photosynthesis as obtained from gas exchange analysis are typically described by the canonical Farquhar–von Caemmerer–Berry (FvCB) biochemical photosynthesis model (Farquhar *et al.*, 1980). This model predicts that leaf photosynthetic rates under the current environmental conditions are determined by two main parameters, Rubisco carboxylation capacity (*V*_cmax_) and maximum linear electron transport rate (*J*_max_). The transition from Rubisco limitation to electron-transport limitation occurs around a leaf internal CO_2_ level when the equivalent ambient-air CO_2_ concentration (*C*_a_) is about 400 μmol mol^−1^ (e.g. Mathan *et al.*, 2021). However, the internal CO_2_ inside a leaf can attain a high level at which leaf photosynthesis is limited by a third parameter, *T*_p_, the rate of triose phosphate utilization (TPU). Until now, compared with the first two biochemical components (i.e. *V*_cmax_ and *J*_max_), *T*_p_ has received less attention since observable TPU limitations only occur occasionally and are highly variable, depending on species, genotype, growth conditions, and measurement conditions (e.g. Kumarathunge *et al.*, 2019). Also, the TPU-limited condition is observed only temporarily, because it can be removed quickly as other parameters like *V*_cmax_ and *J*_max_ may be regulated to a level where *T*_p_ is no longer ‘apparently’ limiting (McClain *et al.*, 2023). Nevertheless, with the increase of atmospheric CO_2_ (to roughly 600 μmol mol^−1^; Lombardozzi *et al.*, 2018), TPU limitation will probably become increasingly important for predicting photosynthesis and yield.

TPU refers to the rate at which triose phosphates exit from the photosynthetic Calvin–Benson–Bassham (CBB) cycle and are used as sugar precursors for processes like the synthesis of sucrose and starch. As triose phosphates are phosphorylated carbon, any TPU requires the returns of inorganic phosphate (P_i_) to the chloroplast, since the quantity of phosphate in the chloroplast is finite and under tight homeostasis (McClain and Sharkey, 2019). A limitation of TPU on photosynthesis is triggered when carbon exports from (with the accompanying P_i_ import to) the cycle cannot keep pace with carbon fixation, which is in essence a local sink–source disequilibrium. TPU limitation causes unresponsiveness to CO_2_ (Sharkey, 1985) or sometimes reversed sensitivity of photosynthesis to increasing CO_2_ (e.g. von Caemmerer and Farquhar, 1981; Harley and Sharkey, 1991). The TPU limitation is considered to be a biochemical mechanism for sink limitation on photosynthesis, expressed at a sub-foliar scale (Sharkey, 2019). This differs from the sink (panicles) limitation on source (leaves) activity agronomists commonly define at the whole-plant or crop scale. While sink limitations at sub-foliar and whole-plant scales are not necessarily independent (Yin *et al.*, 2022), few studies have been conducted on their connections.

Agronomists commonly manipulate sink–source ratios at the whole-plant scale by pruning leaves or panicles during the grain filling process, which modifies the transport of assimilates between source and sink organs (Li *et al.*, 2017; He *et al.*, 2019). Many reports (Rossi *et al.*, 2015; Dingkuhn *et al.*, 2020; Fabre *et al.*, 2020) have also demonstrated that genotypes with larger crop carbon sink capacity can benefit more from future CO_2_-rich climate. Conversely, a smaller sink (e.g. small-panicle genotypes, or plants with panicles pruned) reduces phloem loading, forcing assimilate accumulation in leaves or stems that may exert a feedback inhibition on leaf photosynthetic source activity (Burnett *et al.*, 2016; White *et al.*, 2016), and even on TPU (Fabre *et al.*, 2019).

Carbon metabolism generally interacts with nitrogen (N) assimilation. In fact, Harley & Sharkey (1991) hypothesized that the reversed sensitivity of photosynthesis to increasing CO_2_ under TPU limitation resulted from diverting a fraction of N-containing glycine from the photorespiratory pathway and this glycine is used elsewhere for other amino acid or protein synthesis. Glycine is derived from the photorespiratory glycolate carbon; normally 25% of the glycolate carbon is lost as CO_2_ as a result of glycine decarboxylation, and the remaining 75% is recycled to glycerate and further to 3-phosphoglycerate to rejoin the CBB cycle (Supplementary Fig. S1). With the exit of glycine, the P_i_ normally used in converting glycerate to 3-phosphoglycerate is made available for phosphorylation, thereby stimulating photosynthesis (Harley and Sharkey, 1991). Busch *et al.* (2018) extended this hypothesis by considering the exit of both glycine and serine from the photorespiratory pathway. The export of N from the photorespiratory pathway requires *de novo* N assimilation and amino acid synthesis (Supplementary Fig. S1). They proposed that a large proportion of N assimilation in leaves accomplished via the photorespiratory pathway is innately linked with TPU. The exported amino acids represent an additional sink for carbon and decrease P_i_ consumption for phosphorylating glycerate to 3-phosphoglycerate, thereby explaining the increased photosynthetic rate with decreasing CO_2_ levels (with increasing photorespiration) within the TPU-limited range (Busch *et al.*, 2018; Yin *et al.*, 2021). The extent of increase in photosynthesis depends on the proportion of glycolate carbon exported from the photorespiratory pathway. It is conceivable that the proportion of amino acid carbon export may be associated with the availability of NO_3_^−^ for N assimilation. Crop N assimilation occurs throughout the life cycle, but varies with NO_3_^−^ availability and leaf N content. We hypothesize that values of a parameter related to TPU limitation (i.e. the fraction of the glycolate carbon exported from the photorespiratory pathway) increase with leaf N content, and thus vary among growth stages and N treatments.

In this study, we aim to (i) quantify how TPU-limited photosynthetic carbon uptake is affected by nitrogen assimilation via the photorespiratory pathway; and (ii) analyse how the local sink–source mechanisms of TPU limitation within leaves are regulated by the whole-plant physiological source and sink relationships. We address these aims in the context of our recent effort to examine the impact of leaf-colour modification on photosynthesis, using rice genotypes of different leaf colour based on our previous finding that genotypic leaf yellowness affects leaf photosynthetic rate via several mechanisms including altered leaf morphology (Zhou *et al.*, 2023). As adaxial and abaxial photosynthetic rates are known to differ (e.g. Soares *et al.*, 2008), gas exchange measurements with the local illumination on either leaf side may result in a varied leaf-scale source activity but with a constant sink demand. Thus, in our experimental set-up, we use three means to vary carbon sink–source ratios (genotypic leaf-colour variants, panicle pruning, and adaxial versus abaxial illumination while measuring gas exchange), and two means to vary nitrogen status (N treatments and plant developmental stages). In addition, we use 21% O_2_ versus 2% O_2_ conditions to alter photorespiration, likely modifying the amount of glycine and serine export. We hoped to obtain information on how TPU limitation is related to photorespiration-associated N assimilation and affected by altered sink–source ratios within the leaf and at the whole-plant scale.

## Materials and methods

### Plant material and growth conditions

Rice (*Oryza sativa* L.) materials were based on two background genotypes: japonica type cv. Wuyunjing 3 (WYJ) and early indica type cv. Zhefu 802 (ZF). Both were modified by radiation mutagenesis with ^60^Co γ-rays, and the yellower-leaf variants were identified from a larger population of phenotypes. These genotypes showed stability of the lines over generations (Zhou *et al.*, 2023). Hereafter, yellower genotypes are denoted as Y and the wild type as control (C).

Two experiments were conducted in a climate-controlled glasshouse in Wageningen, the Netherlands in 2019 and 2022. The growth conditions were the same as described in our earlier study (Zhou *et al.*, 2023): incident global radiation outside the greenhouse was kept within 400−500 W m^−2^ (resulting in a photosynthetic photon flux density measured at plant height of ~500 μmol m^−2^ s^−1^), temperature was set at 26 °C for the 12-h light period and at 23 °C for the 12-h dark period, the CO_2_ level was about 400 μmol mol^−1^, and the relative humidity was 65−75%. The 2019 experiment was to examine if genotypes differ in the extent of photosynthetic differences between adaxial and abaxial illumination.

In the 2022 experiment, nitrogen supply and panicle pruning treatments were added as factors. Nitrogen was applied as urea at two levels: N1 (in total 0.7 g urea per pot) and N2 (in total 1.4 g urea per pot). All pots were evenly divided into four blocks (corresponding to four experimental replicates), and each block contained 64 pots, representing all combinations of four genotypes, two nitrogen levels, and two pruning levels. The four pots per treatment combination were used for measurements at three developmental stages (see below), with one pot as reserve in case of plant damage. Plants to be pruned were randomly selected and pre-labelled, and panicles of these plants were pruned at the moment when the first panicle of the plant had emerged from the flag-leaf sheath. This operation lasted a week to ensure that no new heads were produced from any culms.

### Leaf photosynthesis measurements

Pre-labelled and fully expanded main-stem leaves in each experimental treatment per replicate were measured using an open-path gas exchange system integrated with a fluorescence chamber head (LI-COR 6800; LI-COR Inc., Lincoln, NE, USA) to simultaneously obtain gas exchange and chlorophyll fluorescence parameters. All measurements were carried out at a leaf temperature of 25 °C and a vapour pressure difference of 1.0−1.6 kPa between the leaf and air outside of the leaf, with a flow rate of 400 μmol s^−1^.

For both 2019 and 2022 experiments, measurements were conducted on the same leaf segment at both adaxial and abaxial sides. It should be noted that measurements on ‘adaxial’ or ‘abaxial’ sides always integrated the gas exchange occurring on both sides, as both sides were exposed to the chamber air. However, the light was only received by the side that faced the light source. Strictly speaking, it was not the gas exchange measurement but the light orientation that varied, causing inverted light gradients through the leaf.

For the 2019 experiment, measurements were conducted only at the tillering stage. Light and CO_2_ response curves were measured at the same position at both adaxial and abaxial sides of the leaves. The curves for net photosynthetic rate (*A*) response to incident irradiance (*I*_inc_) were obtained with *I*_inc_ in a decreasing series of 2000, 1500, 1000, 500, 280, 150, 100, 80, and 50 μmol m^−2^ s^−1^ (6−8 min per step), while maintaining ambient CO_2_ level (*C*_a_) at 400 μmol mol^−1^. The CO_2_ response curves were measured at *I*_inc_ of 1000 μmol m^−2^ s^−1^, with the *C*_a_ steps of 400, 250, 150, 80, 50, 400, 400, 400, 650, 1000, and 1500 μmol mol^−1^ (3−5 min per step; note that using the three repeated 400 μmol mol^−1^ steps was merely to re-adapt leaves, and the data from these three points were excluded in the analysis). Both curves were measured at ambient O_2_ (21%) level. To estimate day respiration (*R*_d_) and establish a calibration factor (*s*) that converts chlorophyll fluorescence-based electron transport efficiency of photosystem II (PSII) into linear electron transport rate (see Yin *et al.*, 2009), we also conducted half of the light response curve (with *I*_inc_ being 280, 150, 100, 80, and 50 μmol m^−2^ s^−1^) under non-photorespiratory conditions (2% O_2_ combined with *C*_a_ at 1000 μmol mol^−1^). These low light levels were applied to ensure that data for calibration were within the range where *A* is limited by electron transport. The low O_2_ level was realized by using a cylinder containing a gas mixture of 2% O_2_ and 98% N_2_.

For the 2022 experiment, photosynthesis was measured on both adaxial and abaxial leaf surfaces at three stages: tillering stage (TS), flowering stage (FS), and grain-filling (~15 days after flowering (DAF)). The panicle-pruned plants were only measured at grain filling because these plants were supposed to function the same as the non-pruned plants at tillering and flowering. As this experiment was meant to examine the TPU limitation, only CO_2_ response curves (where *A* is likely limited by TPU) were measured at *I*_inc_ of 1500 μmol m^−2^ s^−1^ under both 21% and 2% O_2_ conditions, with the *C*_a_ in an increasing series: 400, 500, 600, 700, 800, 900, 1000, 1200, 1400, 1600, and 1800 μmol mol^−1^. These *C*_a_ levels were chosen to ensure that part of the curve could reach the TPU-limited range. As with the 2019 experiment, we additionally measured the light-response curve with *I*_inc_ being 300, 150, 100, 80, and 40 μmol m^−2^ s^−1^ under non-photorespiratory conditions to estimate *R*_d_ and *s*.

For each irradiance or CO_2_ step in both experiments, *F*_s_ (the steady-state fluorescence) was recorded after *A* reached the steady state. The maximum fluorescence (*F*ʹ_m_) was determined using a three-phase flash method (Loriaux *et al.*, 2013): each phase went through a duration of 300 ms, and flash intensity of 6500 μmol m^−2^ s^−1^ in the second phase was attenuated by 40%. The apparent operating photochemical efficiency of PSII was assessed from chlorophyll fluorescence measurements: Φ_2_=1−*F*_s_/*F’*_m_ (Genty *et al.*, 1989).

All gas exchange data were corrected for any small basal leakage of CO_2_ into and out of the leaf cuvette, based on measurements on boiled leaves across the CO_2_ levels, and intercellular CO_2_ levels (*C*_i_) were then re-calculated.

### Leaf SPAD and nitrogen content

All leaf segments used for measuring photosynthesis curves were cut out and used immediately to measure the leaf area with a LI-3100 area meter (LI-COR) and the values for SPAD indicating chlorophyll content (SPAD-502, Minolta Camera Co., Japan). SPAD was measured at both adaxial and abaxial sides of these leaf segments. Leaf materials were then oven-dried at 70 °C for 48 h to constant weight. Specific leaf area (SLA, m^2^ kg^−1^) was calculated as the leaf area to dry leaf mass ratio. Each leaf segment was ground into powder in a 2-ml centrifuge tube, which was used to measure the N concentration by an element analyser based on the micro-Dumas combustion method. Specific leaf nitrogen (SLN, g N m^−2^) was then calculated.

### Plant growth measurements

At grain-filling stage in the 2022 experiment, the aboveground parts were sampled and separated. Dry weight of each part was determined after oven drying at 75 °C for 72 h to constant weight. The leaf samples were ground into powder, which was then assessed for nitrogen concentration with a Kjeldahl apparatus (Kjeltec 8400, Foss Corp., Germany). Total leaf-nitrogen per pot was calculated by leaf nitrogen concentration multiplied by total leaf dry weight. We counted the fertile spikelet number for each culm, and then measured the flag leaf area (just after photosynthesis measurement) and total leaf area by a LI-3100 area meter (LI-COR). Following Fabre *et al.* (2020), the ratio of flag leaf area (source) to the fertile spikelet number of the panicle (sink) on the culm was used as an indicator of the single-culm sink–source ratio, while total spikelet number divided by total leaf area of the whole plant was used as an indicator of the whole-plant sink–source ratio.

### Estimating photosynthetic parameters

We estimated parameters of the FvCB model (Farquhar *et al.*, 1980), which expresses net photosynthetic rate (*A*) as the minimum of the Rubisco carboxylation-limited rate (*A*_c_), electron-transport limited rate (*A*_j_), and the TPU-limited rate (*A*_p_):


$$\begin{array}{l} A=min\left(A_{c},A_{j},A_{p}\right) \end{array}$$ (1)

For *A*_c_:


$$\begin{array}{l} A_{c}=\frac{\left(C_{c}-\Gamma_{*}\right)V_{cmax}}{C_{c}+K_{mC}\left(1+O/K_{mO}\right)}-R_{d} \end{array}$$ (2)

where *C*_c_ and *O* are the chloroplast partial pressures of CO_2_ and O_2_, respectively, *V*_cmax_ is the maximum rate of Rubisco activity for carboxylation, and *K*_mC_ and *K*_mO_ are Michaelis–Menten constants of Rubisco for CO_2_ and O_2_, respectively. Γ_*_ is the CO_2_ compensation point in the absence of day respiration (*R*_d_), described by: Γ_*_=0.5*O*/*S*_c/o_, where *S*_c/o_ is the relative CO_2_/O_2_ specificity factor for Rubisco. Values of these Rubisco parameters vary significantly, depending on techniques used to measure them; here, we used the representative values of Rubisco parameters measured *in vitro* at 25 °C by Cousins *et al.* (2010) for wheat: i.e. 291 μbar for *K*_mC_, 194 mbar for *K*_mO_, and 3.022 mbar μbar^−1^ for *S*_c/o_, given that values of these Rubisco parameters are believed to be conserved among C_3_ species (von Caemmerer, 2000).

For *A*_j_:


$$\begin{array}{l} A_{j}=\frac{\left(C_{c}-\Gamma_{*}\right)J}{4\left(C_{c}+2\Gamma_{*}\right)}-R_{d} \end{array}$$ (3a)

where *J* is the potential linear electron transport rate supporting the CBB cycle and the photorespiratory cycle. *J* can be calculated using the calibration factor *s*, incident irradiance (*I*_inc_), and fluorescence-based photochemical efficiency of PSII (Φ_2_) as: $J=sI_{inc}\Phi_{2}$, where parameters *s* and *R*_d_ can be estimated from the slope and intercept of a linear plot of *A*_j_ against (*I*_inc_Φ_2_/4) measured under non-photorespiratory conditions (Yin *et al.*, 2009). The calculated *J* can be fitted according to:


$$\begin{array}{l} J=\left[\kappa_{2LL}I_{inc}+J_{max}-\sqrt{{\left(\kappa_{2LL}I_{inc}+J_{max}\right)}^{2}-4\theta J_{max}\kappa_{2LL}I_{inc}}\right]/\left(2\theta \right) \end{array}$$ (3b)

where *J*_max_ is the maximum value of *J* under saturated light; κ_2LL_ represents the conversion efficiency of incident light into *J* at strictly limiting light; and θ is a dimensionless convexity factor for the response of *J* to *I*_inc_, and here a common value of 0.76 for θ was adopted for all rice genotypes from Zhou *et al.* (2023).

For *A*_p_, the widely used algorithm (Harley and Sharkey, 1991; von Caemmerer, 2000) assumes that glycine is taken out from the photorespiratory pathway. However, this algorithm does not consider the required change of the CO_2_ compensation point, as a result of the glycine export, to (1−α_G_)Γ_*_ (Busch *et al.*, 2018; Yin *et al.*, 2021, also see Supplementary Fig. S1; where α_G_ is the proportion of glycolate carbon exported from the photorespiratory pathway in the form of glycine). Model fitting results of Busch *et al.* (2018) suggested that the proportion of glycolate carbon exported as glycine is lower than the proportion exported as serine. Isotope-labelling measurements (Abadie *et al.*, 2016; Fu *et al.*, 2022) more convincingly confirmed little export in the form of glycine. As serine export causes no change in the CO_2_ compensation point, here for the purpose of simplicity, we assumed only the serine export, for which the model becomes (Yin *et al.*, 2021; see their Equation 17b):


$$\begin{array}{l} A_{p}=\frac{\left(C_{c}-\Gamma_{*}\right)(3T_{p})}{C_{c}-\left(1+4\alpha_{S}\right)\Gamma_{*}}-R_{d} \end{array}$$ (4)

where α_S_ is the proportion of glycolate carbon exported from photorespiratory pathway in the form of serine (with 0≤α_S_≤0.75). This guarantees the same term in the numerator, (*C*_c_−Γ_*_), which is consistent with Rubisco- or electron transport-limited forms. Such consistency simplifies the modelling algorithms for the next steps of analysis (Equations 5, 6). The simple model in Equation 4 also generates the TPU-limited rate *A*_p_ similar to the full model with both glycine and serine export if the total fraction of glycolate carbon export remains the same (Yin *et al.*, 2021). Note that the coefficient in front of the term for the proportion of glycolate carbon export in the denominator of Equation 4 is 4, whereas this is 3 in the commonly used old equation assuming the glycine exit (von Caemmerer, 2000; Ellsworth *et al.*, 2015; Busch and Sage, 2017; Kumarathunge *et al.*, 2019; also see Supplementary Fig. S1). As a result, the old equation, when applied to fit gas-exchange data, overestimates the glycolate carbon export fraction by a factor of 4/3 (Yin *et al.* 2021).

The method of Harley *et al.* (1992) was first applied to examine whether mesophyll conductance *g*_m_ varied with intercellular CO_2_ level (*C*_i_) or *I*_inc_, and we found that *g*_m_ is variable and declines with increasing *C*_i_ or with decreasing *I*_inc_, with *g*_m_=0 as *I*_inc_ approaches to zero (results not shown). To describe this pattern of variable *g*_m_, we used an equation of Yin *et al.* (2009):


$$\begin{array}{l} g_{m}=\delta \left(A+R_{d}\right)/\left(C_{c}-\Gamma_{*}\right) \end{array}$$ (5)

where parameter δ represents the carboxylation resistance to mesophyll resistance ratio (Yin *et al.*, 2020). Then, this Equation 5 was combined with Equations 2, 3a, and 4, and *C*_c_ was replaced by (*C*_i_−*A*/*g*_m_) to solve for *A* (Yin *et al.*, 2020):


$$\begin{array}{l} A=\left(-b\pm \sqrt{b^{2}-4ac}\right)/\left(2a\right) \end{array}$$ (6)

where


$$\begin{array}{l} a=x_{2}+\Gamma_{*}+\delta \left(C_{i}+x_{2}\right) \end{array}$$

$$\begin{array}{lll} & b=-\left(x_{2}+\Gamma_{*}\right)\left(x_{1}-R_{d}\right)-\delta \left(C_{i}+x_{2}\right)\left(x_{1}-R_{d}\right)-\; & \delta \left[x_{1}\left(C_{i}-\Gamma_{*}\right)-R_{d}\left(C_{i}+x_{2}\right)\right] \end{array}$$

$$\begin{array}{l} c=\delta \left(x_{1}-R_{d}\right)\left[x_{1}\left(C_{i}-\Gamma_{*}\right)-R_{d}\left(C_{i}+x_{2}\right)\right] \end{array}$$

where


$$\begin{array}{l} For\;A_{c}\;part\;\;\;\left\{ \begin{array}{l} x_{1}=V_{cmax}\;x_{2}=K_{mC}\left(1+O/K_{mO}\right)\; \end{array}\right.\;\;\; \end{array}$$

$$\begin{array}{l} For\;\;\;A_{j}\;part\;\;\;\left\{ \begin{array}{l} x_{1}=J/4\;x_{2}=2\Gamma_{*}\; \end{array}\right.\;\;\;\;\; \end{array}$$

$$\begin{array}{l} For\;\;\;A_{p}\;part\;\;\;\;\;\;\;\left\{ \begin{array}{l} x_{1}=3T_{p}\;x_{2}=-\left(1+4\alpha_{S}\right)\Gamma_{*}\; \end{array}\right.\;\;\;\;\; \end{array}$$

Note when calculating *A* in Equation 6, the minus sign in front of the $\sqrt{b^{2}-4ac}\;$ term was applied for either the *A*_c_- or *A*_j_-limited part while the + sign was required for *A*_p_-limited part (see Yin *et al.*, 2020).

For analysing the 2019 data where light-response curves were measured, we first estimated *J*_max_ by fitting equation 3b to data points of light response of *J* derived from chlorophyll fluorescence parameters Φ_2_ (i.e. *J*=*sI*_inc_Φ_2_). Then photosynthetic parameters δ, *V*_cmax_, *T*_p_, and α_S_ can be estimated simultaneously by fitting combined Equations 1, 3b, and 6 to all CO_2_ exchange data from both light- and CO_2_-response curves. For data from 2022, as only CO_2_ response curves were measured yet starting with *C*_a_ from 400 μmol mol^−1^ onwards that only covered *A*_j_- and *A*_p_-limited parts, we thus combined *J*=*sI*_inc_Φ_2_ and *A*_j_ and *A*_p_ parts of Equation 6 to estimate parameters δ, *T*_p_, and α_S_ simultaneously.

Once photosynthetic parameters were estimated, the transition point from *A*_j_- to *A*_p_-limited rates can be solved. We also estimated the transition point by solving the second-order polynomial regression equations that were fitted to *A*_j_ and *A*_p_ ranges, respectively, of *A*–*C*_i_ curves. The estimated threshold *C*_i_ was highly consistent (see Results); thus, we used the polynomial-based values for showing the transition.

### Statistical analyses and curve fitting

Simple linear regressions were conducted using Microsoft Excel. Non-linear regressions were performed using the Gauss method in PROC NLIN of SAS (SAS Institute Inc., Cary, NC, USA). An analysis of variance (ANOVA) of multiple experimental factors (i.e. genotype, adaxial versus abaxial, pruning, N level, stage), and their interaction effects on each parameter was performed in the 2022 experiment. A multiple comparison of means was then performed using the LSD (least significant difference) test.

## Results

### Effect of adaxial versus abaxial measurements on leaf source activity

**Fig. 1.**
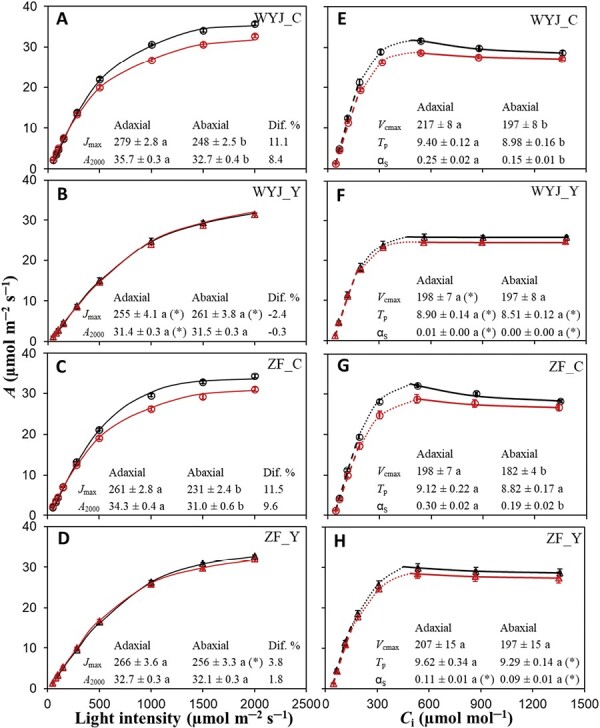
Effects of adaxial versus abaxial measurement on the photosynthetic parameters in four rice genotypes (data measured at the tillering stage in the 2019 experiment). (A–D) Light-response curves of photosynthesis (*A*) at the CO_2_ concentration of 400 μmol mol^─1^. (E–H) CO_2_-response curves at the light intensity of 1000 μmol m^─2^ s^─1^, for rice control (C) genotypes (circles) and their yellower-leaf (Y) variant genotypes (triangles). Data shown as the mean of four replicates (±SE) for each genotype, with black symbols representing measurement on adaxial surface of leaves and red symbols representing measurement on abaxial surface of leaves. WYJ and ZF are the abbreviations of two genetic backgrounds Wuyunjing 3 and Zhefu 802, respectively. For (A–D), the curves are drawn from Equation 6 using fitted parameter values. The estimated maximum linear electron transport under saturating light (*J*_max_, μmol m^─2^ s^─1^), net photosynthesis rate under light intensity of 2000 μmol m^─2^ s^─1^ (*A*_2000_, μmol m^─2^ s^─1^), and the percentage difference in the *J*_max_ and *A*_2000_ (calculated as [(Adaxial−Abaxial)/Adaxial]×100) are listed. For (E–H), the curves representing *A*_c_- (dashed curve), *A*_j_- (dotted curve), and *A*_p_-limited (full curve) parts are drawn from Equation 6 using fitted values of the parameters: the estimated maximum rate of Rubisco carboxylation (*V*_cmax_, µmol m^─2^ s^─1^), rate of triose phosphate utilization (*T*_p_, µmol m^─2^ s^─1^), and the proportion of glycolate carbon exported from the photorespiratory pathway in the form of serine (α_S_). The different letters indicate statistical significance at the *P*<0.05 level for the estimated parameters between adaxial and abaxial measurements, and the asterisks represent significant differences (*P*<0.05) between C genotype and its Y variant.

In the 2019 experiment, the two Y-variant genotypes exhibited an opposite trend in leaf photosynthetic rate (*A*): relative to their control (C) genotypes, a decrease in *A* and estimated parameters (*J*_max_, *V*_cmax_, and *T*_p_) was obtained in WYJ-Y whereas an increase in these parameters was observed in ZF-Y (Fig. 1). In addition, the parameter α_S_ was also altered by the Y modification: it became lower (*P*<0.05), particularly in the WYJ background.

The difference in photosynthetic rates between the two sides of the same leaf depended on genotypes. For light response curves (Fig. 1A–D), a great reduction (ca. 8−10%) in *A* at a light intensity of 2000 μmol m^−2^ s^−1^ (*A*_2000_) was observed on the abaxial side in C genotypes, resulting in a lower estimated *J*_max_ on the abaxial surface compared with that on the adaxial surface. In contrast, the light response curves were similar on both sides of leaves in Y-variant genotypes (difference of *A*_2000_ less than 2%). Similar patterns were observed for CO_2_ response curves (Fig. 1E–H), with greater differences between adaxial and abaxial values for parameters *V*_cmax_, *T*_p_, and α_S_ in C genotypes than in Y-variant genotypes.

### Overview of *A–C*_*i*_ curves from the 2020 experiment

Given the above differences between adaxial and abaxial measurements in the 2019 experiment, we continued in the 2022 experiment to use adaxial versus abaxial measurements for all nitrogen×pruning combinations as a means to manipulate within-leaf sink–source ratios. In addition, measurements were made for three different stages and at two O_2_ levels. All the *A*–*C*_i_ curves obtained are shown in Supplementary Fig. S2.

Differences in measured *A*–*C*_i_ curves and in the estimated *A*_j_-to-*A*_p_ transition point between 21% and 2% O_2_ (Supplementary Fig. S2) agreed with those theoretically expected for TPU limitation either with (Supplementary Fig. S2A) or without (Supplementary Fig. S2B) glycolate carbon exit from the photorespiratory pathway (Harley and Sharkey, 1991; Busch *et al.,* 2018). Thus, combined data from the two O_2_ levels were fit to estimate TPU parameters.

### Estimated triose phosphate utilization capacity

**Fig. 2.**
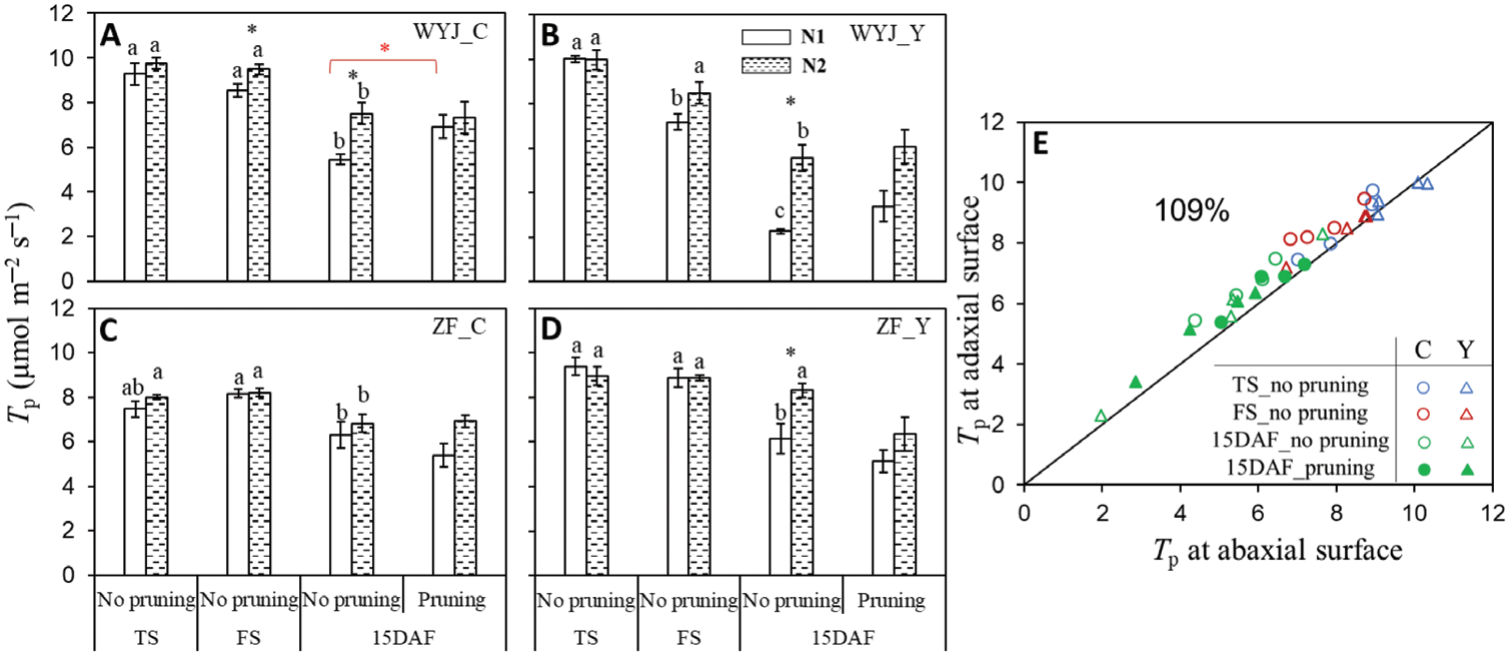
Effects of altered sink–source ratios on parameter *T*_p_ (the 2022 experiment). (A–D) The rate of triose phosphate utilization (*T*_p_, based on measurements on the adaxial leaf surface) for rice control (C) genotypes and their yellower-leaf (Y) variant genotypes of intact or panicle-pruned plants at tillering (TS), flowering (FS), and 15 days after flowering (DAF) stages under low-nitrogen (N1, white bars) and high-nitrogen (N2, dashed bars) levels. The value of each bar representing the mean ±SE of four replicates was estimated by fitting curves to CO_2_ exchange data (see Supplementary Fig. S2). For intact plants (no pruning), different letters indicate statistical significance at the *P*<0.05 level between three stages within each genotype–nitrogen combination, and the asterisks in black represent significant differences (*P*<0.05) between N1 and N2 levels within each genotype and stage. The asterisk in red represents significant difference (*P*<0.05) for a given genotype–nitrogen combination between pruned and un-pruned plants at 15 DAF stage. WYJ and ZF are the abbreviations of two genetic backgrounds: cv. Wuyunjing 3 and cv. Zhefu 802. (E) Comparisons of the values of *T*_p_ measured at adaxial surface versus those measured at abaxial surface. The percentage is the average of adaxial relative to abaxial parameters and the diagonal line is the 1:1 line. Data represented by different colours and symbols are from C genotypes (circles) and Y-variant genotypes (triangles) of intact (open symbols) and panicle-pruned (filled symbols) plants at TS (blue), FS (red), and 15 DAF (green) stage. Each point represents the mean of three or four replicates.

Values of *T*_p_ estimated for pruned and non-pruned plants under two nitrogen levels (N1 and N2) and at three growth stages in the 2022 experiment are shown in Fig. 2. As expected, the rate of TPU (*T*_p_; Fig. 2A–D; Supplementary Table S1) and photosynthetic rate at a light intensity of 1500 μmol m^−2^ s^−1^ (*A*_1500_; Supplementary Table S2) increased with the addition of N fertilizer and decreased with advancing growth stage. Significant effects mainly occurred from flowering onwards. At 15 DAF, there were no significant effects of panicle pruning on *T*_p_ except for an increase of *T*_p_ in WYJ-C at N1 (Fig. 2A). In line with the results of the 2019 experiment (Fig. 1), values of *T*_p_ from the adaxial measurements were generally higher than those from the abaxial measurements (Fig. 2E).

### Estimated proportion of photorespiratory carbon exited as serine

**Fig. 3.**
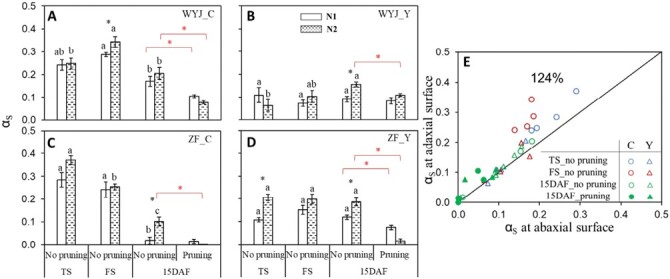
Effects of various growth stages and nitrogen levels on parameter α_S_, the proportion of glycolate carbon exported from the photorespiratory pathway in the form of serine (the 2022 experiment). (A–D) α_S_ (based on measurements on the adaxial leaf surface) for rice control (C) genotypes and their yellower-leaf (Y) variant genotypes of intact or panicle-pruned plants at tillering (TS), flowering (FS), and 15 days after flowering (DAF) stages under low-nitrogen (N1, white bars) and high-nitrogen (N2, dashed bars) levels. The value of each bar representing the mean ±SE of four replicates was estimated by fitting curves to CO_2_ exchange data (see Supplementary Fig. S2). For intact plants (no pruning), different letters indicate statistical significance at the *P*<0.05 level between three stages within each genotype–nitrogen combination, and the asterisks in black represent significant differences (*P*<0.05) between N1 and N2 levels within each genotype and stage. The asterisks in red represent significant differences (*P*<0.05) for a given genotype-nitrogen combination between pruned and un-pruned plants at 15 DAF stage. WYJ and ZF are the abbreviations of two genetic backgrounds: cv. Wuyunjing 3 and cv. Zhefu 802. (E) Comparison of the values of α_S_ measured at adaxial surface versus those measured at abaxial surface. The percentage is the average of adaxial relative to abaxial parameters and the diagonal line is the 1:1 line. Data represented by different colours and symbols are from C genotypes (circles) and Y-variant genotypes (triangles) of intact (open symbols) and panicle-pruned (filled symbols) plants at TS (blue), FS (red), and 15 DAF (green) stage. Each point represents the mean of three or four replicates.

The effects of N treatments on the values of α_S_ are shown in Fig. 3. The high N level generally increased α_S_ (*P*<0.05). But growth-stage effects were more complex, as α_S_ varied more among growth stages in C genotypes than in Y variants (Fig. 3A–D). The estimated α_S_ of WYJ-C declined significantly (*P*<0.05) at 15 DAF after an increase at FS, whereas that of ZF-C decreased along all growth stages. The estimated α_S_ was negatively correlated with SLA, an indicator of leaf thinness (Supplementary Fig. S3).

Unlike *T*_p_, α_S_ was greatly decreased by pruning in all genotypes, especially at N2. There was a significant interaction between pruning and N level on α_S_ (*P*<0.001; Supplementary Table S3). In addition, compared with *T*_p_, α_S_ differed more between adaxial and abaxial measurements (by ~24%), with larger differences in C genotypes than in Y-variant genotypes, especially at TS and FS stages (Fig. 3E). No interactions of measurement side with N level or growth stage were found for α_S_ (Supplementary Table S3).

### Correlations between triose phosphate utilization parameters and leaf nitrogen content

**Fig. 4.**
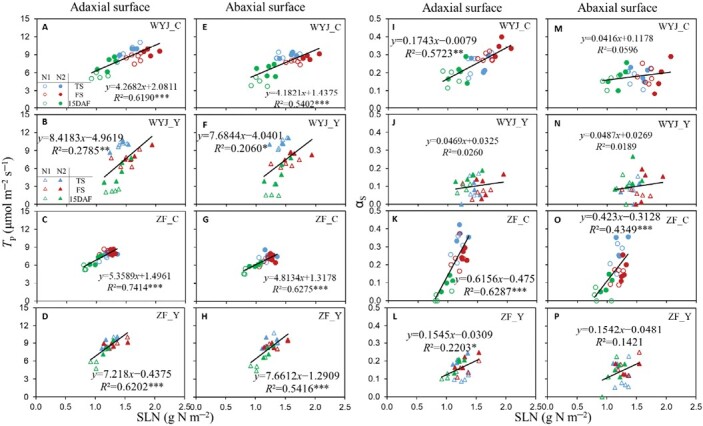
Relationship between photosynthetic parameters and leaf nitrogen content (based on measurements on the un-pruned plants in the 2022 experiment). (A–H) Relationship between triose phosphate utilization rate (*T*_p_) and specific nitrogen content (SLN). (I–P) Relationship between the proportion of glycolate carbon exported from photorespiratory pathway in the form of serine (α_S_) and SLN. Data represented by different colours and symbols are from tillering (TS, blue), flowering (FS, red), and 15 days after flowering (DAF) (green) stage under low-nitrogen (N1, open symbols) and high-nitrogen (N2, filled symbols) levels, with circles for rice control (C) genotypes and triangles for their yellower-leaf (Y) variant genotypes. Linear regressions were fitted for each genotype with four or five replicates across two nitrogen levels and three stages. The significance of each correlation is shown by asterisks: *P<0.05, **P<0.01, ***P<0.001. WYJ and ZF are the abbreviations of two genetic backgrounds: cv. Wuyunjing 3 and cv. Zhefu 802.

In general, *T*_p_ and α_S_ were positively correlated with SLN across all N levels and growth stages, but correlations were genotype-dependent (Fig. 4). Given the smaller α_S_ values in Y genotypes (Figs 1, 3), slopes were smaller and correlations were poorer, particularly when measuring the abaxial leaf side, compared with the C genotypes.

**Fig. 5.**
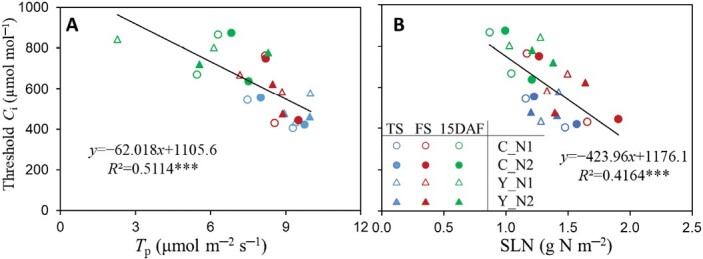
The threshold *C*_i_ values in relation to leaf physiological parameters. (A) Relationship between threshold *C*_i_ and the rate of triose phosphate utilization (*T*_p_) (data based on measurements on the adaxial surface of leaves in the 2022 experiment). (B) Relationship between threshold *C*_i_ and specific leaf nitrogen (SLN). The threshold *C*_i_ represents the transition point where the photosynthesis-limiting process changed from electron transport to TPU, derived from CO_2_ response curves (see Supplementary Fig. S2). Data represented by different colours and symbols are from tillering (TS, blue), flowering (FS, red), and 15 days after flowering (DAF) (green) stage under low-nitrogen (N1, open symbols) and high-nitrogen (N2, filled symbols) levels, with circles for rice control (C) genotypes and triangles for their yellower-leaf (Y) variant genotypes. Linear regressions were fitted for overall data with the significance of each correlation shown by asterisks: ***P<0.001.

The threshold *C*_i_ values (at which TPU became limiting) estimated by the two methods were highly consistent (Supplementary Fig. S4). The threshold *C*_i_ increased with advancing growth stage. It was negatively correlated with *T*_p_ (*R*^2^=0.51, *P*<0.001; Fig. 5A) and with SLN (*R*^2^=0.42, *P*<0.001; Fig. 5B).

### Triose phosphate utilization-limited photosynthesis in relation to whole-plant sink limitation

**Fig. 6.**
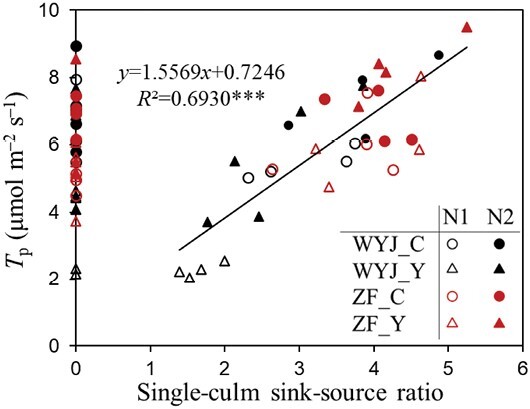
Relationship between the rate of triose phosphate utilization (*T*_p_, based on measurements on the adaxial leaf surface) and single-culm sink–source ratio. Here, following Fabre *et al.* (2020), the ratio of flag leaf area (source) to the fertile spikelet number of the panicle (sink) on the culm was used as an indicator of the single-culm sink–source ratio (also see the text). Data are for rice control (C) genotypes (circles) and yellower-leaf (Y) variant genotypes (triangles) from grain-filling stage under low-nitrogen (N1, open symbols) and high-nitrogen (N2, filled symbols) levels in the 2022 experiment, with cv. Wuyunjing 3 (WYJ) in black and cv. Zhefu 802 (ZF) in red. For those plants with panicle pruning, we define their sink–source ratio to be zero, so all their data points fall on the *y*-axis. Linear regression was fitted for data (representing no pruning) with the significance of the correlation shown by asterisks: ***P<0.001.

**Fig. 7.**
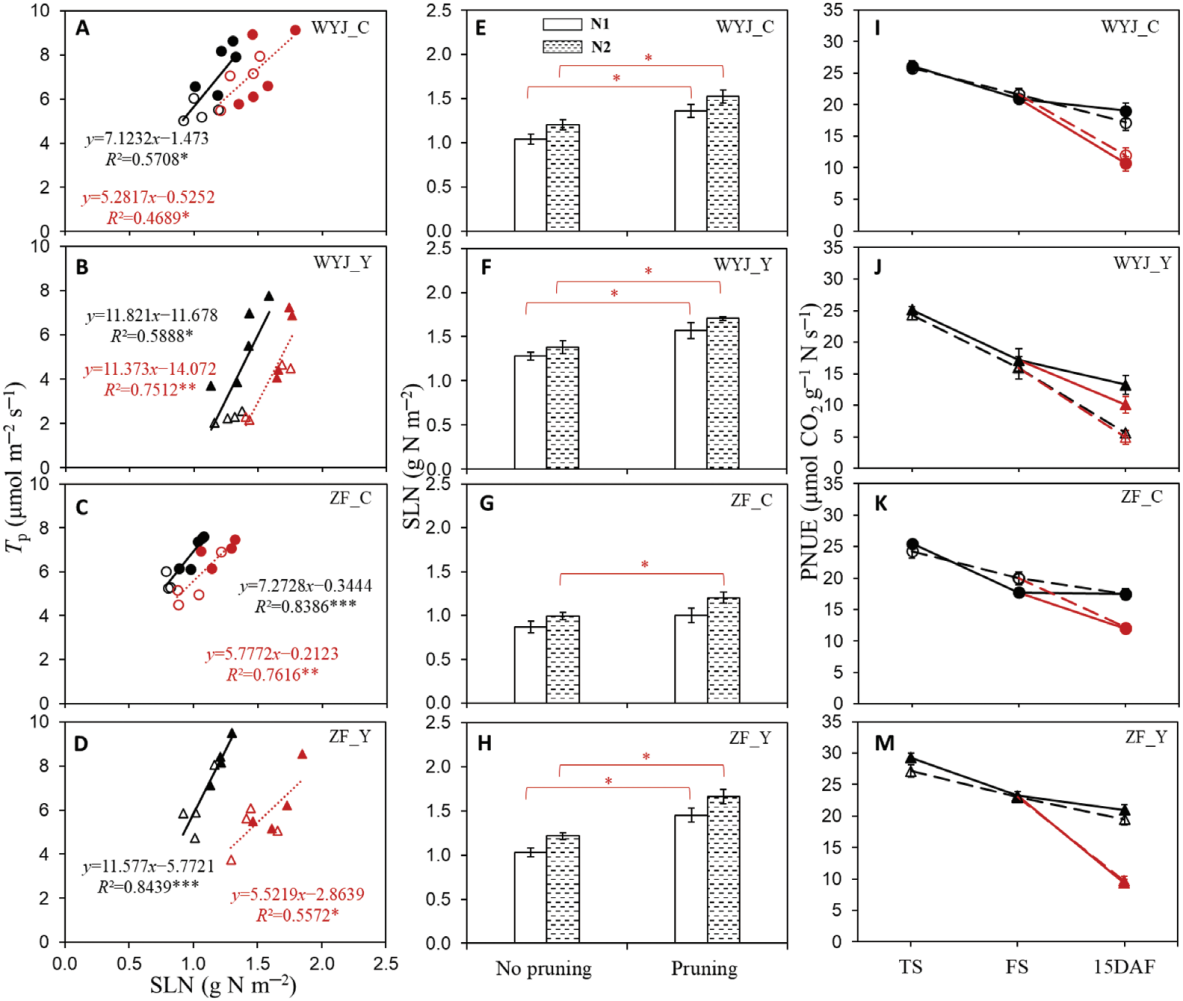
The effects of panicle pruning on leaf photosynthetic parameters (based on measurements on the adaxial leaf surface in the 2022 experiment). (A–D) Relationship between the rate of triose phosphate utilization (*T*_p_) and specific leaf nitrogen (SLN) for the intact and panicle-pruned plants. (E–H) Effect of panicle pruning on SLN. (I–M) Leaf photosynthetic nitrogen-use efficiency (PNUE) at tillering (TS), flowering (FS), and 15 days after flowering (DAF) stage (see Supplementary Table S2 for the definition of PNUE). Linear regressions in (A–D) were fitted for each genotype with four or five replicates under two nitrogen levels; the significance of each correlation is shown by asterisks: *P<0.05, **P<0.01, ***P<0.001. Data in (E–M) represent the mean ±SE of four replicates; the asterisks represent significant differences (*P*<0.05) within each genotype–nitrogen combination between unpruned and pruned plants. The data in (A–H) are from the 15 DAF stage, and the data in (A–D) and (I–M) represent the values for rice control (C) genotypes (circles) and their yellower-leaf (Y) variant genotypes (triangles) of the intact (black) and panicle-pruned (red) plants under low-nitrogen (N1, open symbols) and high-nitrogen (N2, filled symbols) levels. WYJ and ZF are the abbreviations of two genetic backgrounds: cv. Wuyunjing 3 and cv. Zhefu 802.

The variation in *A*_1500_ of each genotype–nitrogen combination, either across stages or across pruning levels, was positively correlated with *T*_p_ for adaxial (Supplementary Fig. S5A) or abaxial surfaces (Supplementary Fig. S5B) of leaves. A positive linear correlation of *T*_p_ with single-culm sink–source ratio (*R*^2^=0.69; Fig. 6) or whole-plant sink–source ratio (*R*^2^=0.60; Supplementary Fig. S6) was observed at the grain-filling stage. Note that data points presented on the line *x*=0 in these figures for panicle-pruned plants (thus, panicle sink was zero) were in a similar range of *T*_p_ (ca. 2–9 μmol m^−2^ s^−1^) of those plants without pruning. This suggests that, overall, there was little observable effect of panicle pruning on *T*_p_. We then compared the correlation between *T*_p_ and SLN for non-pruned and panicle-pruned plants separately (Fig. 7A–D). The pruned plants still had close *T*_p_ correlations with SLN, but the relationship deviated from (e.g. the slope became smaller in most cases) that of the non-pruned plants.

Sink limitation caused by panicle pruning also exerted significant impacts on whole-plant traits, such as increased leaf area, leaf and stem dry weight, and total leaf nitrogen (Table 1). As a result of increased N accumulation in the flag leaf (Fig. 7E–H), panicle pruning lowered leaf photosynthetic nitrogen-use efficiency (PNUE) for each combination of genotype and N level (Fig. 7I–M; *P*<0.001, Supplementary Table S3).

**Table 1.** Values (means ±SE among four replicates) of rice sink and source organs for single culm and whole plant in the control (C) genotypes and the yellower-leaf variant (Y) genotypes at 15 d after flowering stage in the 2022 experiment.

			Single culm		Whole plant				
N level	Background	Genotype	Spikelets per culm (no.)	Flag leaf area (cm^2^)	Total spikelet (no.)	Leaf area (cm^2^ pot^─1^)	Leaf dry weight (g)	Stem dry weight (g)	Total leaf nitrogen (g N pot^─1^)
No pruning									
N1	WYJ	C	47 ± 5	15.5 ± 0.7	543 ± 49	1259.5 ± 27.8	4.8 ± 0.1	17.7 ± 0.8	0.098 ± 0.003
	WYJ	Y	30 ± 3	18.1 ± 0.7	336 ± 24	1361.5 ± 73.0	4.3 ± 0.3	8.2 ± 0.5	0.130 ± 0.007
	ZF	C	101 ± 9	28.7 ± 4.6	811 ± 21	1400.8 ± 59.0	7.7 ± 0.5	34.6 ± 0.9	0.082 ± 0.006
	ZF	Y	114 ± 7	29.7 ± 4.1	893 ± 55	2158.3 ± 97.5	9.4 ± 0.6	19.4 ± 1.2	0.168 ± 0.008
N2	WYJ	C	70 ± 14	17.8 ± 2.8	827 ± 55	1597.7 ± 30.7	6.2 ± 0.1	23.5 ± 1.5	0.129 ± 0.004
	WYJ	Y	46 ± 6	19.7 ± 1.4	797 ± 86	1665.5 ± 86.3	5.9 ± 0.3	12.2 ± 1.0	0.186 ± 0.006
	ZF	C	105 ± 5	26.7 ± 2.7	1324 ± 23	1898.0 ± 50.1	9.0 ± 0.6	34.9 ± 1.1	0.100 ± 0.009
	ZF	Y	132 ± 12	31.2 ± 4.1	1421 ± 54	2702.5 ± 50.8	11.6 ± 0.6	21.8 ± 1.9	0.232 ± 0.020
Pruning									
N1	WYJ	C	—	13.3 ± 2.1	—	1304.6 ± 43.4	5.6 ± 0.1	24.8 ± 1.0	0.106 ± 0.005
	WYJ	Y	—	15.7 ± 1.2	—	1569.5 ± 33.7	5.8 ± 0.2	15.5 ± 1.1	0.179 ± 0.005
	ZF	C	—	29.1 ± 2.4	—	1684.0 ± 76.4	10.5 ± 0.6	43.9 ± 2.6	0.101 ± 0.007
	ZF	Y	—	28.2 ± 3.4	—	2324.6 ± 97.0	10.5 ± 0.5	25.2 ± 1.5	0.186 ± 0.009
N2	WYJ	C	—	19.3 ± 2.2	—	1633.3 ± 31.5	7.1 ± 0.1	28.1 ± 0.3	0.150 ± 0.002
	WYJ	Y	—	17.3 ± 1.3	—	1736.9 ± 86.3	6.6 ± 0.3	16.5 ± 1.3	0.247 ± 0.009
	ZF	C	—	30.9 ± 2.7	—	2240.9 ± 70.5	14.7 ± 0.3	58.6 ± 0.7	0.145 ± 0.004
	ZF	Y	—	35.3 ± 2.5	—	3174.8 ± 88.1	14.3 ± 0.3	27.2 ± 0.8	0.288 ± 0.010
Analysis of variance									
Genotype			46.9***	33.0***	56.9***	214.2***	235.7***	392.6***	167.1***
N level			6.9*	3.6	135.5***	167.8***	114.4***	43.9***	171.4***
Panicle pruning			—	0.1	—	34.4***	105.8***	177.0***	72.8***
Genotype×N level			0.5	0.7	2.1	8.9***	5.2**	3.6*	8.5***
Genotype×panicle pruning			—	0.7	—	3.58*	14.5***	17.7***	4.3**
N level×panicle pruning			—	1.6	—	0.6	6.2*	2.8	7.4**
Genotype×N level×panicle pruning			—	0.2	—	1.8	4.4**	10.4***	0.6

In the analysis of variance, the significance is shown by asterisks: *P<0.05, **P<0.01, ***P<0.001, according to the LSD test. Note, we use ‘genotype’ rather than ‘Y modification’ as a fixed factor mainly because Y modification produces different effects on leaf photosynthetic physiology between WYJ (cv. Wuyunjing 3) and ZF (cv. Zhefu 802) backgrounds (see Results).

## Discussion

In response to sink–source (im)balance, plants can adjust physiological processes at different scales, and these scales can be inter-connected involving dynamic feedback. We hypothesized that the sub-foliar sink–source (im)balance involving triose phosphate utilization (TPU) is regulated by whole-plant sink–source relationships. We used yellower-leaf (Y) modification and adaxial versus abaxial illumination, and 21% versus 2% O_2_ gas mixture to alter the leaf-scale source activity. Panicle pruning was used to alter whole-plant sink/source ratios. The factorial design involving these two scales enabled linking them. By observing plants under different N supply conditions and at three growth stages, we introduced additional variation of source and sink capacity and enabled the establishment of parameter relationships, notably including TPU-limited photosynthesis and photorespiration-associated N assimilation.

### Adaxial versus abaxial measurements on leaf-colour genotypes as a means to alter leaf source activity

In the 2019 experiment using rice genotypes of contrasting leaf colour, a TPU limitation on leaf photosynthesis was observed (Fig. 1). The yellower-leaf variants (Y) differed from their control (C) genotypes in photosynthetic capacity (i.e. *A*_2000_) and underlying parameters (*J*_max_, *V*_cmax_, and *T*_p_), and the difference was mostly expressed when comparing response curves measured under adaxial versus abaxial illumination (Fig. 1). Zhou *et al.* (2023) demonstrated differences in leaf photosynthetic capacity between C and Y genotypes were associated with intra-leaf photosynthetic N reallocation of the surplus N resources liberated by decreased investment in chlorophyll. However, modifying leaf colour changed not only intra-leaf N partitioning but also leaf morphology, such as increased SLA (i.e. thinner leaves) in Y-genotypes (Supplementary Table S2).

This genotypic difference in leaf thickness probably contributed to our result that when light illuminated the abaxial surface, the decrease in leaf photosynthetic capacity parameters was small in Y genotypes but greater in C genotypes (Fig. 1). This suggests that our C genotypes, like plants in previous reports (e.g. Soares *et al.*, 2008), had an adaptive advantage to the adaxial illumination, which is the predominant condition occurring in the field. As the carbon sinks of the leaf and the whole plant were unchanged, altered leaf photosynthetic capacity via adaxial versus abaxial illumination during gas exchange measurement will alter the sink–source ratio, and this was particularly the case in the C genotypes.

### Effects of altered sink–source ratios on leaf triose phosphate utilization limitation

The occurrence of the TPU limitation requires a high photosynthetic rate (Yang *et al.*, 2016). We chose a saturating light intensity (1500 μmol m^−2^ s^−1^) and CO_2_ levels up to 1800 μmol mol^−1^ for measuring *A*–*C*_i_ curves in the 2022 experiment. In our study, *A*_1500_ was highly correlated with *T*_p_ in all cases (Supplementary Fig. S5), reflecting a parallel change between them. Y modification reduced leaf *T*_p_ and *A*_1500_ in WYJ background but increased them in ZF background (Fig. 2A–D; Supplementary Tables S1, S2); adaxial illumination gave higher *T*_p_ than abaxial, especially for C genotypes (Fig. 2E; Supplementary Table S1). Strong correlations between *T*_p_ and SLN in each genotype (Fig. 4) suggested that N always exerted a positive effect on *T*_p_. We also found that the higher the leaf N content (resulting in higher *A*_1500_), the lower was the threshold *C*_i_ where leaf photosynthesis became TPU-limited (Supplementary Fig. S2), as shown by the negative relationship between the threshold *C*_i_ and *T*_p_ or SLN (*P*<0.001; Fig. 5). This relationship was built from data across three growth stages, reflecting dynamic changes in the TPU limitation during rice development.

During the vegetative phase, there is no sink demand from panicles or grains, and all assimilates are used for vegetative organ growth (i.e. absence of a dominant sink like the panicle). In this phase, growing leaves rich in N resources serve as both source and sink organs. The high photosynthetic potential of young plants feeds a plastic (partly facultative) demand exerted by organ development, probably explaining why TPU limitation occurred at a lower *C*_i_ level (ca. 400 μmol mol^−1^; Fig. 5). After flowering, carbon assimilates are exclusively used to support grain growth, and N resources are mobilized from vegetative organs, particularly from leaves (Sinclair and de Wit, 1975). The resulting decrease in *A*_1500_ and *T*_p_ (Fig. 2; Supplementary Tables S1, S2), coupled with increased sink demand, alleviated the extent of TPU limitation.

Our study provides insights on how altered sink–source relationships influence *T*_p_ and thus leaf source activity. The estimated *T*_p_ and the *C*_i_ threshold for the onset of TPU limitation were associated with leaf N content across growth stages. Thus, leaf N content not only determines leaf photosynthetic capacity (Nakano *et al.*, 1995), but also modulates TPU limitation in response to sink–source imbalance.

### Nitrogen assimilation increases photosynthesis under potential triose phosphate utilization limitation

Assimilating NO_3_^−^ via exporting glycolate carbon from the photorespiratory pathway in the form of amino acids can contribute to photosynthetic carbon uptake (Busch *et al.*, 2018; Busch, 2020; Fu *et al.*, 2022). Our *A*–*C*_i_ curves, indicating *A* was higher under 21% O_2_ than under 2% O_2_ conditions, confirmed this—although this advantage from photorespiration diminished with advancing growth stages (Supplementary Fig. S2). The increase in *A* largely depends on the extent of glycolate carbon exported from the photorespiratory pathway (Bauwe *et al.*, 2010; Busch *et al.* 2018; Yin *et al.* 2021; also see Supplementary Fig. S2A, B). Fu *et al.* (2022) demonstrated that the carbon flow out of the pathway was primarily in the form of serine, with a proportion of 23–41% in tobacco plants. Our estimates of parameter α_S_ from model analysis (up to 0.37; Supplementary Table S1) are in line with the measured values of Fu *et al.* (2022) as well as with the modelling results of Busch *et al.* (2018). Based on modelled α_S_, we further assessed the effect of various experimental factors on photorespiration-associated N assimilation. We found that similar to the effect on *T*_p_, leaf N content is also critical to α_S_, as evidenced by correlations between α_S_ and SLN (Fig. 4I–P). However, this positive effect of N level disappeared under Y modification (Supplementary Table S3), since both Y genotypes had lower α_S_ than their corresponding C genotypes (Figs 1, 3; Supplementary Table S1). In conjunction with this, there was a negative relationship between α_S_ and SLA (Supplementary Fig. S3), suggesting that the thinner leaves associated with Y modification may have limited NO_3_^−^ assimilation. This limiting effect was enhanced by illuminating the abaxial surface of leaves during measurements (Fig. 3E; Supplementary Table S3).

In our experiment, urea was used as the N source in fertilizing plants. Busch *et al.* (2018) found that α_S_ was higher in sunflower plants fertilized with NO_3_^−^-N than those with NH_4_^+^-N. Whether this is also the case when applied to our rice plants of different leaf colours remains to be investigated. Previous studies (Reich *et al.*, 2006; Bloom *et al.*, 2012, 2014) reported that elevated growth CO_2_ (resulting in increased source) inhibited the NO_3_^−^ assimilation in C_3_ plants. Here, we found a similar result with a significant drop in α_S_ for the plants after panicle pruning (resulting in decreased sink) (Fig. 3). However, the underlying mechanisms might differ. Elevated growth CO_2_ not only decreased photorespiration but also diluted leaf N concentration via increased biomass (Yin, 2013; Igarashi *et al.*, 2021). In contrast, panicle pruning resulted in an increased N content in leaves (Table 1; Fig. 7E–H). Nevertheless, despite higher leaf N, a lower α_S_ (thus, probably less N assimilation) was observed in leaves of panicle-pruned plants, suggesting a dominating role of whole-plant sink–source ratio in the control of leaf N metabolism.

### Feedback inhibition of whole-plant sink limitation on leaf photosynthesis

It has long been observed that the plant sink–source ratio can affect leaf photosynthesis (Tanaka and Fujita, 1974; Crafts-Brandner and Egli, 1987; Arp, 1991; Li *et al.*, 2015; Aslani *et al.*, 2020). In line with this, studies indicated that a larger sink capacity can increase the effect of elevated atmospheric CO_2_ on photosynthesis and yield (Erice *et al.*, 2011; Hasegawa *et al.*, 2013; Rossi *et al.*, 2015; Kikuchi *et al.*, 2017; Dingkuhn *et al.* 2020). Our finding of a positive correlation between *T*_p_ and the sink–source ratio for single culm (Fig. 6) or for the whole plant (Supplementary Fig. S6) confirmed the role of sink size in modulating source activity of leaves. The reduced *T*_p_ under sink limitation is probably associated with the accumulation of sucrose in leaf photosynthetic tissues (Fabre *et al.* 2019, 2020). This may give a signal regulating the activity of sucrose-phosphate synthase (SPS) through transduction of SnRK1 protein kinases (Halford and Hey, 2009), and the SPS feedback inhibition on sucrose synthesis can decrease export of triose phosphates from the chloroplast (McClain and Sharkey, 2019). Our results also showed that panicle pruning dramatically increased the dry matter accumulation in leaves and stems (Table 1).

Given these considerations, one would expect that for the 15 DAF stage, panicle-pruned plants would have lower *T*_p_ compared with intact plants where the TPU limitation was alleviated. Surprisingly, distinct from previous findings (Fabre *et al.*, 2019; Nomura *et al.*, 2022), *T*_p_ was little affected under this apparently sink-limited state (Figs 2, 6). In the study of Fabre *et al.* (2019), elevated CO_2_ (increased source) coupled with pruning treatment (decreased sink) imposed a dual effect on *T*_p_, which might contribute to more significant sink limitation and feedback effect in their study than in our study. However, the mechanism behind this difference could also be attributed to a larger amount of N accumulated in the leaves of our panicle-pruned plants (Fig. 7E–H). After removal of all panicles, no N was required to be remobilized from leaves to support grain growth and more N remained in leaves. Leaf N is the most important resource for the photosynthetic machinery (Nakano *et al.*, 1995), as shown by our close relationship between *T*_p_ and SLN, even in pruned plants (Fig. 7A–D). Thus, we posit that the feedback inhibition of sink limitation on *T*_p_ still occurred in our panicle-pruned plants, but the inhibitory effects from sink limitation were offset by the increased SLN as a consequence of the smaller N remobilization from leaves, thereby resulting in no observable impact of pruning on *T*_p_ in leaves. The increased SLN did not result in higher N assimilation as α_S_ was lower in panicle-pruned plants (Fig. 3; Supplementary Table S1); as a result, these plants might not benefit from amino acid export for higher CO_2_ uptake rates, but instead, had similar or lower *A*_1500_ compared with the control plants (Supplementary Table S2). Because of the increased SLN (Fig. 7E–H) accompanied with no or little increase in *A*_1500_, leaf PNUE decreased in the panicle-pruned plants (Fig. 7I–M). Taking these together, our results suggest that *T*_p_ and leaf photosynthesis during grain filling are controlled by the whole-plant sink demand and N budgets.

### Concluding remarks

We have shown that the differences in photosynthetic rates between adaxially and abaxially illuminated leaves and the extent of such differences depends on leaf colours of the genotypes. Based on this finding, we further assessed how TPU limitation of photosynthesis, involving photorespiration-associated N assimilation, can be affected by altered sink–source ratios at different scales. We found:

(i) Higher leaf N (observed at early growth stages or high N inputs) caused TPU limitation at relatively low intercellular CO_2_ concentration.
(ii) The proportion of photorespiratory glycolate-carbon exported as serine (α_S_) was positively correlated with leaf N content, suggesting that photorespiration was involved in leaf N assimilation more in high-N than low-N leaves. However, values of α_S_ were smaller in yellower-leaf genotypes, under illumination of abaxial leaf sides, and when panicles were removed.
(iii) Absence of observable effect of panicle pruning on the rate of TPU (*T*_p_) suggested that the feedback inhibition of photosynthesis by whole-plant sink limitation was offset by the positive effect of increased leaf N content, caused by lesser N remobilization.

Thus, our results revealed the crucial role of leaf N in affecting photosynthetic parameters *T*_p_ and α_S_, and thus TPU limitation of photosynthesis. Likewise, N resources modulate the link between leaf-level TPU limitation and whole-plant sink limitation during rice grain filling. The latter link could be masked by the whole-plant N budget, providing a contributing factor (additional to what has been stated in Introduction) about why the sink feedback on, and TPU limitation to, leaf photosynthesis cannot always be observed experimentally. This also adds the complication of disentangling the interaction and causality between observed *A*_1500_ and *T*_p_ in relation to leaf N content. Our results have important implications for modelling crop production in response to a future high-CO_2_ environment, where a delicate balance between source and sink in plants becomes increasingly altered, leaf photosynthesis is expected to be increasingly limited by TPU, and plant N resources tend to be diluted by the greater biomass (Dingkuhn *et al.*, 2020). This warrants more research for better understanding of the N regulatory mechanism in this context so as to effectively screen adaptive traits in rice genotypes for improved crop productivity and nutritional value under futural climatic conditions.

## Supplementary data

The following supplementary data are available at *JXB* online.

Fig. S1. The photorespiratory pathway (involving chloroplast, peroxisome, and mitochondrion), and its connection with the Calvin–Benson–Bassham (CBB) cycle and nitrogen (N) assimilation (revised from Busch, 2020).

Fig. S2. CO_2_-response curves for rice control (C) genotypes and their yellower-leaf (Y) variant genotypes, on both sides of the leaves at three stages, under 21% O_2_ and 2% O_2_ conditions.

Fig. S3. Relationship between the proportion of glycolate carbon exported from photorespiratory pathway in the form of serine (α_S_, based on measurements on the adaxial leaf surface) and specific leaf area (SLA).

Fig. S4. Comparisons of the threshold *C*_i_ (intercellular CO_2_ levels) derived from two methods under adaxial and abaxial measurements.

Fig. S5. Relationship between light-saturated leaf photosynthesis rate (*A*_1500_) and the rate of triose phosphate utilization (*T*_p_) based on adaxial and abaxial measurements.

Fig. S6. Relationship between the *T*_p_ (based on measurements on the adaxial leaf surface) and whole-plant sink–source ratio.

Table S1. Modelled photosynthetic parameters for rice control (C) genotypes and their yellower-leaf (Y) variant genotypes at three stages under low-nitrogen (N1) and high-nitrogen (N2) levels measured from both sides of the leaves in the 2022 experiment.

Table S2. Leaf photosynthetic characteristics for rice control (C) genotypes and their yellower-leaf (Y) variant genotypes at three stages under low-nitrogen (N1) and high-nitrogen (N2) levels measured from both sides of leaves in the 2022 experiment.

Table S3. Summary of analysis of variance of leaf photosynthetic variables in response to genotype, panicle pruning, abaxial versus adaxial measurements, nitrogen level, three-stages’ measurements, and their interactions.

erad329_suppl_Supplementary_Figure_S1-S6_Table_S1-S3

## Data Availability

All data supporting the findings of this study are available within the paper and within its supplementary data published online.

## References

[CIT0001] Abadie C , Boex-Fontvieille ERA, Carroll AJ, Tcherkez G. 2016. *In vivo* stoichiometry of photorespiratory metabolism. Nature Plants 2, 15220. 27249192 10.1038/nplants.2015.220

[CIT0002] Arp WJ. 1991. Effects of source-sink relations on photosynthetic acclimation to elevated CO_2_. Plant, Cell & Environment 14, 869–875.

[CIT0003] Aslani L , Gholami M, Mobli M, Sabzalian MR. 2020. The influence of altered sink-source balance on the plant growth and yield of greenhouse tomato. Physiology and Molecular Biology of Plants 26, 2109–2123. 33268917 10.1007/s12298-020-00891-2 PMC7688802

[CIT0004] Bauwe H , Hagemann M, Fernie AR. 2010. Photorespiration: players, partners and origin. Trends in Plant Science 15, 330–336. 20403720 10.1016/j.tplants.2010.03.006

[CIT0005] Bloom AJ , Asensio JSR, Randall L, Rachmilevitch S, Cousins AB, Carlisle EA. 2012. CO_2_ enrichment inhibits shoot nitrate assimilation in C_3_ but not C_4_ plants and slows growth under nitrate in C_3_ plants. Ecology 93, 355–367. 22624317 10.1890/11-0485.1

[CIT0006] Bloom AJ , Burger M, Kimball BA, Pinter PJ. 2014. Nitrate assimilation is inhibited by elevated CO_2_ in field-grown wheat. Nature Climate Change 4, 477–480.

[CIT0007] Burnett AC , Rogers A, Rees M, Osborne CP. 2016. Carbon source–sink limitations differ between two species with contrasting growth strategies. Plant, Cell & Environment 39, 2460–2472. 10.1111/pce.12801 27422294

[CIT0008] Busch FA. 2020. Photorespiration in the context of Rubisco biochemistry, CO_2_ diffusion and metabolism. The Plant Journal 101, 919–939. 31910295 10.1111/tpj.14674

[CIT0009] Busch FA , Sage RF. 2017. The sensitivity of photosynthesis to O_2_ and CO_2_ concentration identifies strong Rubisco control above the thermal optimum. New Phytologist 213, 1036–1051. 27768823 10.1111/nph.14258

[CIT0010] Busch FA , Sage RF, Farquhar GD. 2018. Plants increase CO_2_ uptake by assimilating nitrogen via the photorespiratory pathway. Nature Plants 4, 46–54. 29229957 10.1038/s41477-017-0065-x

[CIT0011] Cousins AB , Ghannoum O, Von Caemmerer S, Badger MR. 2010. Simultaneous determination of Rubisco carboxylase and oxygenase kinetic parameters in *Triticum aestivum* and *Zea mays* using membrane inlet mass spectrometry. Plant, Cell & Environment 33, 444–452. 10.1111/j.1365-3040.2009.02095.x 20002330

[CIT0012] Crafts-Brandner SJ , Egli DB. 1987. Sink removal and leaf senescence in soybean: cultivar effects. Plant Physiology 85, 662–666. 16665756 10.1104/pp.85.3.662 PMC1054318

[CIT0013] Dingkuhn M , Luquet D, Fabre D, Muller B, Yin X, Paul MJ. 2020. The case for improving crop carbon sink strength or plasticity for a CO_2_-rich future. Current Opinion in Plant Biology 56, 259–272. 32682621 10.1016/j.pbi.2020.05.012

[CIT0014] Ellsworth DS , Crous KY, Lambers H, Cooke J. 2015. Phosphorus recycling in photorespiration maintains high photosynthetic capacity in woody species. Plant, Cell & Environment 38, 1142–1156. 10.1111/pce.12468 25311401

[CIT0015] Erice G , Sanz-Sáez A, Aranjuelo I, Irigoyen JJ, Aguirreolea J, Avice JC, Sánchez-Díaz M. 2011. Photosynthesis, N_2_ fixation and taproot reserves during the cutting regrowth cycle of alfalfa under elevated CO_2_ and temperature. Journal of Plant Physiology 168, 2007–2014. 21880395 10.1016/j.jplph.2011.07.007

[CIT0016] Fabre D , Dingkuhn M, Yin X, Clément-Vidal A, Roques S, Soutiras A, Luquet D. 2020. Genotypic variation in source and sink traits affects the response of photosynthesis and growth to elevated atmospheric CO_2_. Plant, Cell & Environment 43, 579–593. 10.1111/pce.13693 31961455

[CIT0017] Fabre D , Yin X, Dingkuhn M, Clément-Vidal A, Roques S, Rouan L, Soutiras A, Luquet D. 2019. Is triose phosphate utilization involved in the feedback inhibition of photosynthesis in rice under conditions of sink limitation? Journal of Experimental Botany 70, 5773–5785. 31269202 10.1093/jxb/erz318

[CIT0018] Farquhar GD , von Caemmerer S, Berry JA. 1980. A biochemical model of photosynthetic CO_2_ assimilation in leaves of C_3_ species. Planta 149, 78–90. 24306196 10.1007/BF00386231

[CIT0019] Fu X , Gregory LM, Weise SE, Walker BJ. 2022. Integrated flux and pool size analysis in plant central metabolism reveals unique roles of glycine and serine during photorespiration. Nature Plants 9, 169–178. 36536013 10.1038/s41477-022-01294-9

[CIT0020] Genty B , Briantais JM, Baker NR. 1989. The relationship between the quantum yield of photosynthetic electron transport and quenching of chlorophyll fluorescence. Biochimica et Biophysica Acta, General Subjects 990, 87–92.

[CIT0021] Halford NG , Hey SJ. 2009. Snf1-related protein kinases (SnRKs) act within an intricate network that links metabolic and stress signalling in plants. Biochemical Journal 419, 247–259. 19309312 10.1042/BJ20082408

[CIT0022] Harley PC , Loreto F, Marco GD, Sharkey TD. 1992. Theoretical considerations when estimating the mesophyll conductance to CO_2_ flux by analysis of the response of photosynthesis to CO_2_. Plant Physiology 98, 1429–1436. 16668811 10.1104/pp.98.4.1429 PMC1080368

[CIT0023] Harley PC , Sharkey TD. 1991. An improved model of C_3_ photosynthesis at high CO_2_: reversed O_2_ sensitivity explained by lack of glycerate reentry into the chloroplast. Photosynthesis Research 27, 169–178. 24414689 10.1007/BF00035838

[CIT0024] Hasegawa T , Sakai H, Tokida T, et al. 2013. Rice cultivar responses to elevated CO_2_ at two free-air CO_2_ enrichment (FACE) sites in Japan. Functional Plant Biology 40, 148–159. 32481095 10.1071/FP12357

[CIT0025] He A , Wang W, Jiang G, Sun H, Jiang M, Man J, Cui K, Huang J, Peng S, Nie L. 2019. Source-sink regulation and its effects on the regeneration ability of ratoon rice. Field Crops Research 236, 155–164.

[CIT0026] Igarashi M , Yi Y, Yano K. 2021. Revisiting why plants become N deficient under elevated CO_2_: importance to meet N demand regardless of the fed-form. Frontiers in Plant Science 12, 726186. 34804082 10.3389/fpls.2021.726186 PMC8600045

[CIT0027] Kikuchi S , Bheemanahalli R, Jagadish KSV, Kumagai E, Masuya Y, Kuroda E, Raghavan C, Dingkuhn M, Abe A, Shimono H. 2017. Genome-wide association mapping for phenotypic plasticity in rice. Plant, Cell & Environment 40, 1565–1575. 10.1111/pce.12955 28370170

[CIT0028] Kumarathunge DP , Medlyn BE, Drake JE, Rogers A, Tjoelker MG. 2019. No evidence for triose phosphate limitation of light-saturated leaf photosynthesis under current atmospheric CO_2_ concentration. Plant, Cell & Environment 42, 3241–3252. 10.1111/pce.13639 31378950

[CIT0029] Li G , Pan J, Cui K, Yuan M, Hu Q, Wang W, Mohapatra PK, Nie L, Huang J, Peng S. 2017. Limitation of unloading in the developing grains is a possible cause responsible for low stem non-structural carbohydrate translocation and poor grain yield formation in rice through verification of recombinant inbred lines. Frontiers in Plant Science 8, 1369. 28848573 10.3389/fpls.2017.01369 PMC5550689

[CIT0030] Li T , Heuvelink E, Marcelis LFM. 2015. Quantifying the source-sink balance and carbohydrate content in three tomato cultivars. Frontiers in Plant Science 6, 416. 26097485 10.3389/fpls.2015.00416 PMC4456573

[CIT0031] Lombardozzi DL , Smith NG, Cheng SJ, Dukes JS, Sharkey TD, Rogers A, Bonan GB. 2018. Triose phosphate limitation in photosynthesis models reduces leaf photosynthesis and global terrestrial carbon storage. Environmental Research Letters 13, 074025.

[CIT0032] Loriaux SD , Avenson TJ, Welles JM, Mcdermitt DK, Eckles RD, Riensche B, Genty B. 2013. Closing in on maximum yield of chlorophyll fluorescence using a single multiphase flash of sub-saturating intensity. Plant, Cell & Environment 36, 1755–1770. 10.1111/pce.12115 23586649

[CIT0033] Mathan J , Singh A, Jathar V, Ranjan A. 2021. High photosynthesis rate in two wild rice species is driven by leaf anatomy mediating high Rubisco activity and electron transport rate. Journal of Experimental Botany 72, 7119–7135. 34185840 10.1093/jxb/erab313

[CIT0034] McClain AM , Cruz JA, Kramer DM, Sharkey TD. 2023. The time course of acclimation to the stress of triose phosphate use limitation. Plant, Cell & Environment 46, 64–75. 10.1111/pce.14476 PMC10100259 36305484

[CIT0035] McClain AM , Sharkey TD. 2019. Triose phosphate utilization and beyond: from photosynthesis to end product synthesis. Journal of Experimental Botany 70, 1755–1766. 30868155 10.1093/jxb/erz058 PMC6939825

[CIT0036] Nakano H , Makino A, Mae T. 1995. Effects of panicle removal on the photosynthetic characteristics of the flag leaf of rice plants during the ripening stage. Plant and Cell Physiology 36, 653–659.

[CIT0037] Nomura K , Saito M, Ito M, Yamane S, Iwao T, Tada I, Yamazaki T, Ono S, Yasutake D, Kitano M. 2022. Diurnal decline in the photosynthetic capacity of uppermost leaves in an eggplant canopy grown in a horticultural greenhouse. Photosynthetica 60, 457–464. 10.32615/ps.2022.040 PMC11558575 39650106

[CIT0038] Reich PB , Hobbie SE, Lee T, Ellsworth DS, West JB, Tilman D, Knops JMH, Naeem S, Trost J. 2006. Nitrogen limitation constrains sustainability of ecosystem response to CO_2_. Nature 440, 922–925. 16612381 10.1038/nature04486

[CIT0039] Rossi M , Bermudez L, Carrari F. 2015. Crop yield: challenges from a metabolic perspective. Current Opinion in Plant Biology 25, 79–89. 26002068 10.1016/j.pbi.2015.05.004

[CIT0040] Sharkey TD. 1985. Photosynthesis in intact leaves of C_3_ plants: physics, physiology and rate limitations. The Botanical Review 51, 53–105.

[CIT0041] Sharkey TD. 2019. Is triose phosphate utilization important for understanding photosynthesis? Journal of Experimental Botany 70, 5521–5525. 31624849 10.1093/jxb/erz393 PMC6812704

[CIT0042] Sinclair TR , De Wit CT. 1975. Photosynthate and nitrogen requirements for seed production by various crops. Science 189, 565–567. 17798304 10.1126/science.189.4202.565

[CIT0043] Soares AS , Driscoll SP, Olmos E, Harbinson J, Arrabaça MC, Foyer CH. 2008. Adaxial/abaxial specification in the regulation of photosynthesis and stomatal opening with respect to light orientation and growth with CO_2_ enrichment in the C_4_ species *Paspalum dilatatum*. New Phytologist 177, 186–198. 17850248 10.1111/j.1469-8137.2007.02218.x

[CIT0044] Tanaka A , Fujita K. 1974. Nutrio-physiological studies on the tomato plant IV. Source-sink relationship and structure of the source-sink unit. Soil Science and Plant Nutrition 20, 305–315.

[CIT0045] von Caemmerer S. 2000. Biochemical models of leaf photosynthesis. Collingwood, Victoria, Australia: CSIRO publishing.

[CIT0046] von Caemmerer S , Farquhar GD. 1981. Some relationships between the biochemistry of photosynthesis and the gas exchange of leaves. Planta 153, 376–387. 24276943 10.1007/BF00384257

[CIT0047] White AC , Rogers A, Rees M, Osborne CP. 2016. How can we make plants grow faster? A source-sink perspective on growth rate. Journal of Experimental Botany 67, 31–45. 26466662 10.1093/jxb/erv447

[CIT0048] Yang JT , Preiser AL, Li Z, Weise SE, Sharkey TD. 2016. Triose phosphate use limitation of photosynthesis: short-term and long-term effects. Planta 243, 687–698. 26620947 10.1007/s00425-015-2436-8

[CIT0049] Yin X. 2013. Improving ecophysiological simulation models to predict the impact of elevated atmospheric CO_2_ concentration on crop productivity. Annals of Botany 112, 465–475. 23388883 10.1093/aob/mct016 PMC3718207

[CIT0050] Yin X , Busch FA, Struik PC, Sharkey TD. 2021. Evolution of a biochemical model of steady-state photosynthesis. Plant, Cell & Environment 44, 2811–2837. 10.1111/pce.14070 PMC8453732 33872407

[CIT0051] Yin X , Gu J, Dingkuhn M, Struik PC. 2022. A model-guided holistic review of exploiting natural variation of photosynthesis traits in crop improvement. Journal of Experimental Botany 73, 3173–3188. 35323898 10.1093/jxb/erac109 PMC9126731

[CIT0052] Yin X , Struik PC, Romero P, Harbinson J, Evers JB, Van Der Putten PEL, Vos J. 2009. Using combined measurements of gas exchange and chlorophyll fluorescence to estimate parameters of a biochemical C_3_ photosynthesis model: a critical appraisal and a new integrated approach applied to leaves in a wheat (*Triticum aestivum*) canopy. Plant, Cell & Environment 32, 448–464. 10.1111/j.1365-3040.2009.01934.x 19183300

[CIT0053] Yin X , van der Putten PEL, Belay D, Struik PC. 2020. Using photorespiratory oxygen response to analyse leaf mesophyll resistance. Photosynthesis Research 144, 85–99. 32040701 10.1007/s11120-020-00716-z PMC7113236

[CIT0054] Zhou Z , Struik PC, Gu J, van der Putten PEL, Wang Z, Yin X, Yang J. 2023. Enhancing leaf photosynthesis from altered chlorophyll content requires optimal partitioning of nitrogen. Crop and Environment 2, 24–36.

