# Supplementary material for: Triose phosphate utilization in leaves is modulated by whole-plant sink–source ratios and nitrogen budgets in rice
Source: J Exp Bot. 2023 Aug 29;74(21):6692–707. doi: 10.1093/jxb/erad329 (PMC10662237; doi:10.1093/jxb/erad329)
Supplement: erad329_suppl_Supplementary_Figure_S1-S6_Table_S1-S3 [file erad329_suppl_supplementary_figure_s1-s6_table_s1-s3.pdf]

Supplementary information

Triose phosphate utilisation in leaves is modulated by whole-plant sink-source ratios and nitrogen budgets in rice

Zhenxiang Zhou[1], Zichang Zhang[1,2], Peter E.L. van der Putten[1], Denis Fabre[3,4], Michael Dingkuhn[3,4], Paul C. Struik[1] and Xinyou Yin[1,*]

[1] Centre for Crop Systems Analysis, Department of Plant Sciences, Wageningen University & Research, PO Box 430, 6700 AK Wageningen, The Netherlands

[2] Institute of Plant Protection, Jiangsu Academy of Agricultural Sciences, Nanjing, Jiangsu, China

[3] CIRAD, UMR AGAP Institut, F-34398 Montpellier, France

[4] UMR AGAP Institut, Univ Montpellier, CIRAD, INRAE, Institut Agro, Montpellier, France

[*] Correspondence: xinyou.yin@wur.nl

Contents of this file

Supplementary Figures S1-S6 and Supplementary Tables S1-S3

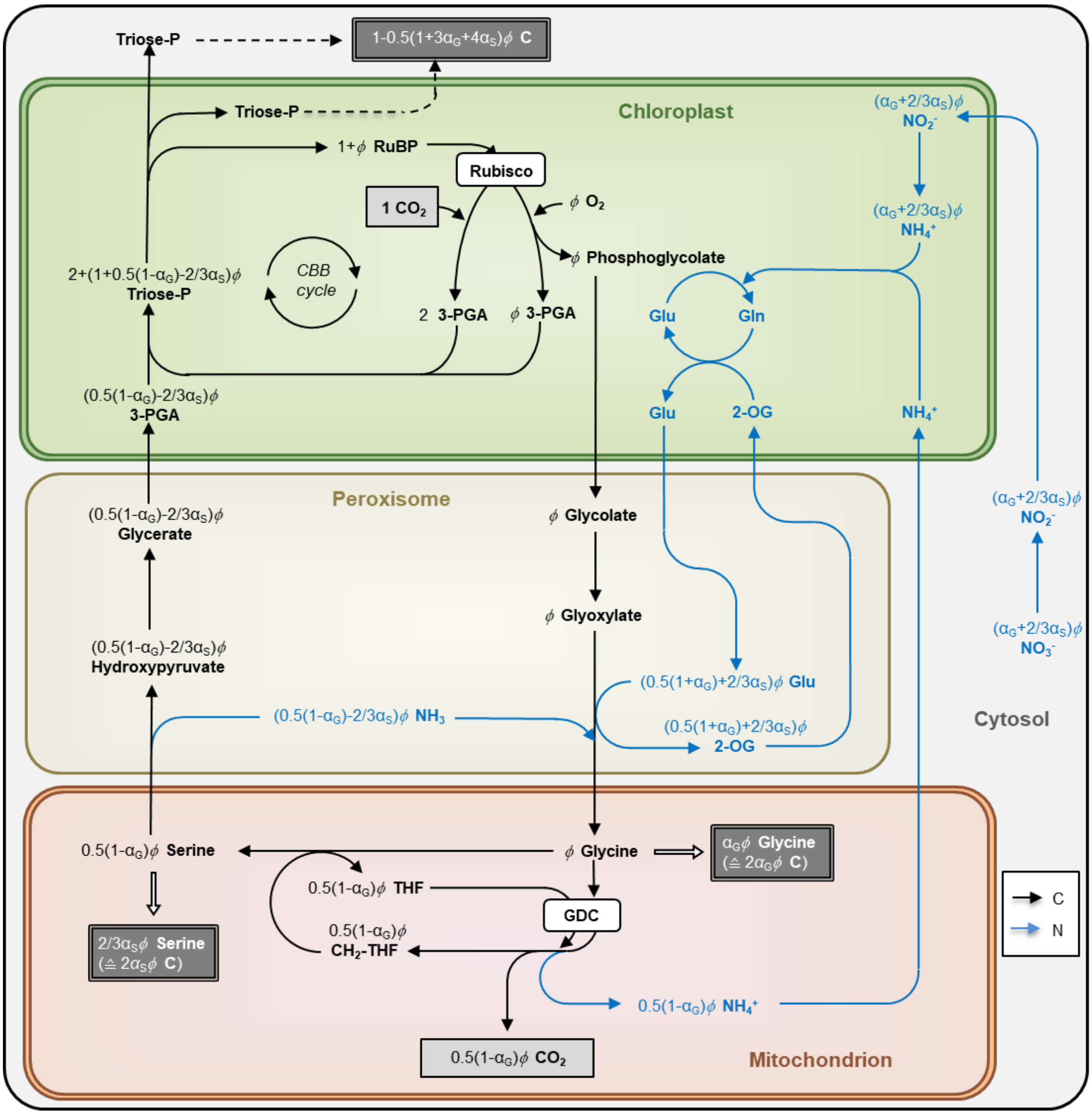

Fig. S1. The photorespiratory pathway (involving chloroplast, peroxisome, and mitochondrion), and its connection with the Calvin-Benson-Bassham (CBB) cycle and nitrogen (N) assimilation (redrawn from Busch, 2020, with permission). The scheme assumes certain fractions of glycolate-carbon exit in the form of either glycine (α_G) or serine (α_S) from the pathway for other uses in plant metabolism. All carbon (in black) and N (in blue) fluxes are scaled relative to the rate of RuBP carboxylation (the first step of the CBB cycle) while the ratio of RuBP oxygenation (the first step of the photorespiratory pathway) to RuBP carboxylation is denoted as ϕ. The amount of NO_3^- needed to enter the leaf via *de novo* N assimilation equals the total flux of glycine- and serine-nitrogen leaving the photorespiratory pathway $(\alpha_G+2/3\alpha_S)\phi$. Regardless of triose-P used as sugar precursors for processes like starch synthesis in chloroplasts or like sucrose synthesis in cytosol (indicated by dashed arrows), the sum of individual sinks for assimilated carbon (indicated by double-bordered black boxes including those for glycine and serine exits) equals CO_2 taken up by RuBP carboxylation minus CO_2 released by glycine decarboxylase (GDC) in the mitochondrion (the source of "photorespiration") indicated by single-bordered grey boxes. Note that the CO_2 release by GDC will be decreased (by a factor of α_G) if glycine exits, whereas this is not the case if serine exits, from the pathway. This difference has implications for the CO_2-compensation point and thus for modelling leaf photosynthesis. For example, TPU-limited photosynthesis (A_p) can be modelled by eqn (4) in the main text if serine exits, whereas the model has to be changed to: $A_p = \frac{[C_c-\Gamma_*(1-\alpha_G)](3T_p)}{C_c-(1+3\alpha_G)\Gamma_*} - R_d$ if glycine exits (Busch et al., 2018; Yin et al., 2021). These two equations give a similar A_p when T_p and the glycolate-carbon exit fraction stay the same, whereas the commonly used algorithm $A_p = \frac{(C_c-\Gamma_*)(3T_p)}{C_c-(1+3\alpha_G)\Gamma_*} - R_d$ underpredicts A_p (Yin et al., 2021). Abbreviations: 2-OG, 2-oxoglutarate; 3-PGA, 3-phosphoglycerate; CH_2-THF, 5,10-methylene-tetrahydrofolate; Gln, glutamine; Glu, glutamate; RuBP, ribulose 1,5-bisphosphate; THF, tetrahydrofolate; triose-P, triose phosphate.

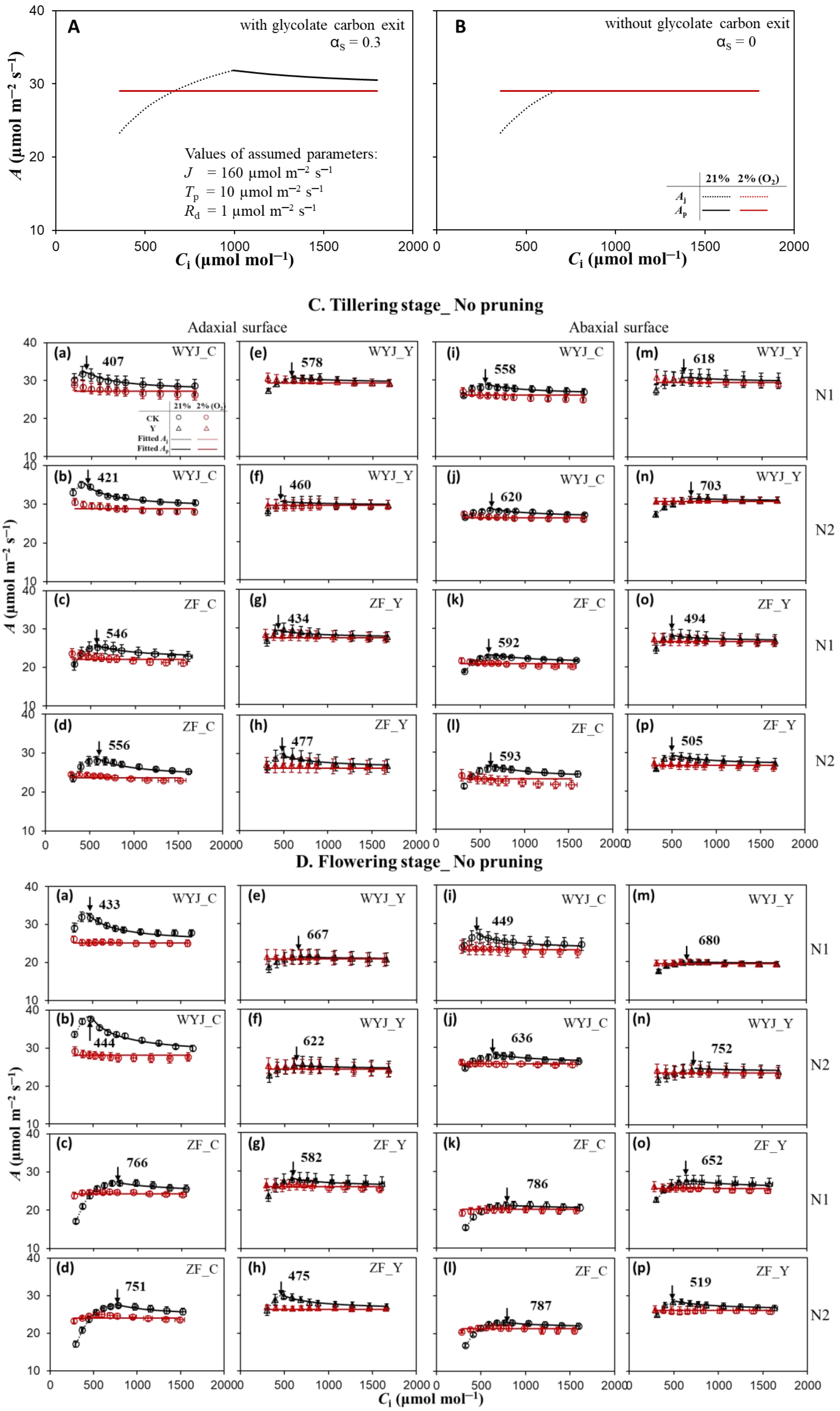

A
with glycolate carbon exit
αS = 0.3
Values of assumed parameters:
J = 160 µmol m−2 s−1
Tp = 10 µmol m−2 s−1
Rd = 1 µmol m−2 s−1
B
without glycolate carbon exit
αS = 0
21% 2% (O2)
Aj
Ap
A (µmol m−2 s−1)
Ci (µmol mol−1)
C. Tillering stage_ No pruning
Adaxial surface
Abaxial surface
(a) 407 WYJ_C
(e) 578 WYJ_Y
(i) 558 WYJ_C
(m) 618 WYJ_Y
N1
CK
Y
Fitted Aj
Fitted Ap
(b) 421 WYJ_C
(f) 460 WYJ_Y
(j) 620 WYJ_C
(n) 703 WYJ_Y
N2
(c) 546 ZF_C
(g) 434 ZF_Y
(k) 592 ZF_C
(o) 494 ZF_Y
N1
(d) 556 ZF_C
(h) 477 ZF_Y
(l) 593 ZF_C
(p) 505 ZF_Y
N2
D. Flowering stage_ No pruning
(a) 433 WYJ_C
(e) 667 WYJ_Y
(i) 449 WYJ_C
(m) 680 WYJ_Y
N1
(b) 444 WYJ_C
(f) 622 WYJ_Y
(j) 636 WYJ_C
(n) 752 WYJ_Y
N2
(c) 766 ZF_C
(g) 582 ZF_Y
(k) 786 ZF_C
(o) 652 ZF_Y
N1
(d) 751 ZF_C
(h) 475 ZF_Y
(l) 787 ZF_C
(p) 519 ZF_Y
N2
Ci (µmol mol−1)

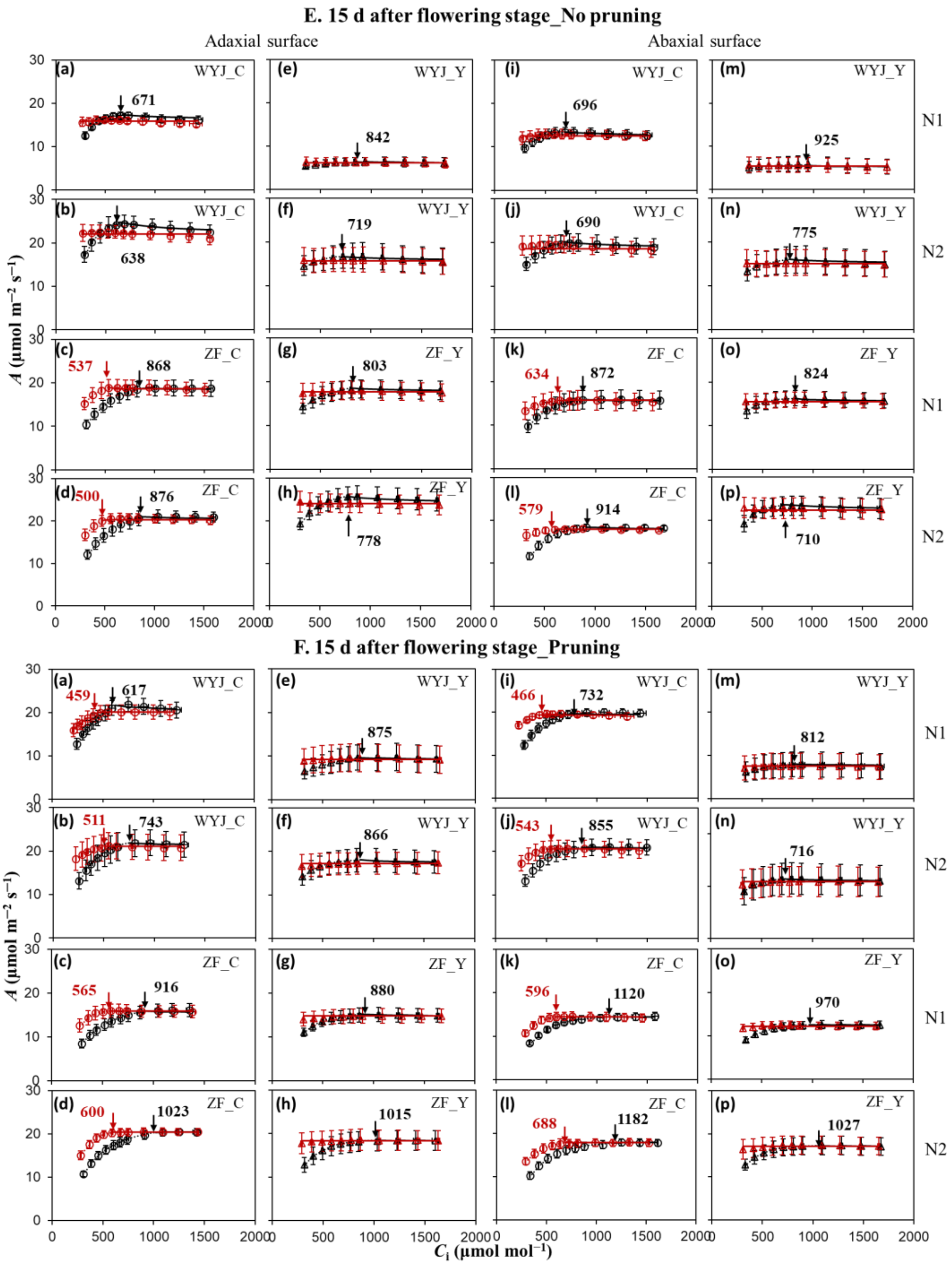

Fig. S2. A-C_i curves with black and red representing trends under 21% O_2 and 2% O_2 conditions, respectively. **A-B,** Theoretical curves with (A; $\alpha_S = 0.3$) or without (B; $\alpha_S = 0$) glycolate carbon exit from the photorespiratory pathway, drawn using assumed parameters (listed in Panel A; note that in Panel B, the black curve under 21% O_2 within the range of TPU limitation is invisible, because it is overlapped by the red one under 2% O_2). **C-F,** Measured A-C_i curves in the 2022 experiment for rice control (C) genotypes (circles) and their yellower-leaf (Y) variant genotypes (triangles), on both sides of the leaves at three stages. Data are shown as means of four replicates (± standard errors) for each genotype. The curves are drawn from eqn (6) using fitted parameter values (see Table S1), representing fitted A_j (dotted lines) and A_p (full lines). The transition points (termed "threshold C_i", see Fig. 6) shown with arrows (black for 21% and red for 2% O_2 conditions) in each panel are the intersection points of A_j and A_p curves (those red curves without an arrow means that the TPU limitation was already reached at a lower C_i out of the measured range). WYJ and ZF are the abbreviations of two genetic backgrounds: cv. Wuyunjing 3 and cv. Zhefu 802.

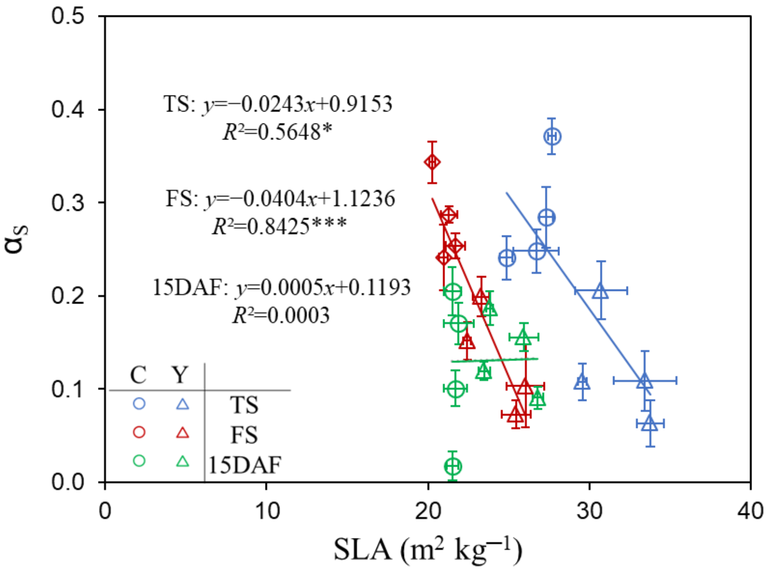

Fig. S3. Relationship between the proportion of glycolate carbon exported from photorespiratory pathway in the form of serine (α_S, based on measurements on the adaxial leaf surface) and specific leaf area (SLA). Data represented by different colours and symbols are the values for rice control (C) genotypes (circles) and yellower-leaf (Y) variant genotypes (triangles) of these un-pruned plants from tillering (TS, blue), flowering (FS, red), and 15 d after flowering (15DAF, green) stage in the 2022 experiment. Each point represents the mean of three or four replicates. Linear regressions were fitted for each stage with the significance of each correlation shown by asterisks: * $P < 0.05$, *** $P < 0.001$.

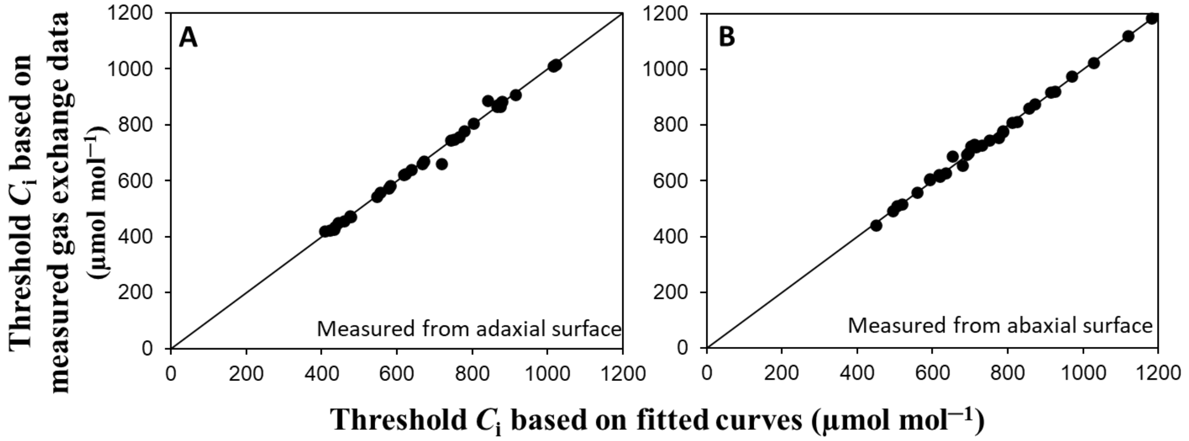

Fig. S4. Comparisons of the threshold C_i derived from two methods under adaxial (A) and abaxial (B) measurements (data based on the 2022 experiment). The first method for threshold C_i (the *y*-axis) was solved as the intersection point of second-order polynomial regression ($y=ax^2+bx+c$) equations that best fitted to measured gas exchange data representing A_j- and A_p-ranges. The second method for threshold C_i (the *x*-axis) was obtained from the extrapolated intersection point of A_j- and A_p-part fitted curves drawn from eqn (6) based on the estimated parameters. The diagonal line is the 1:1 line.

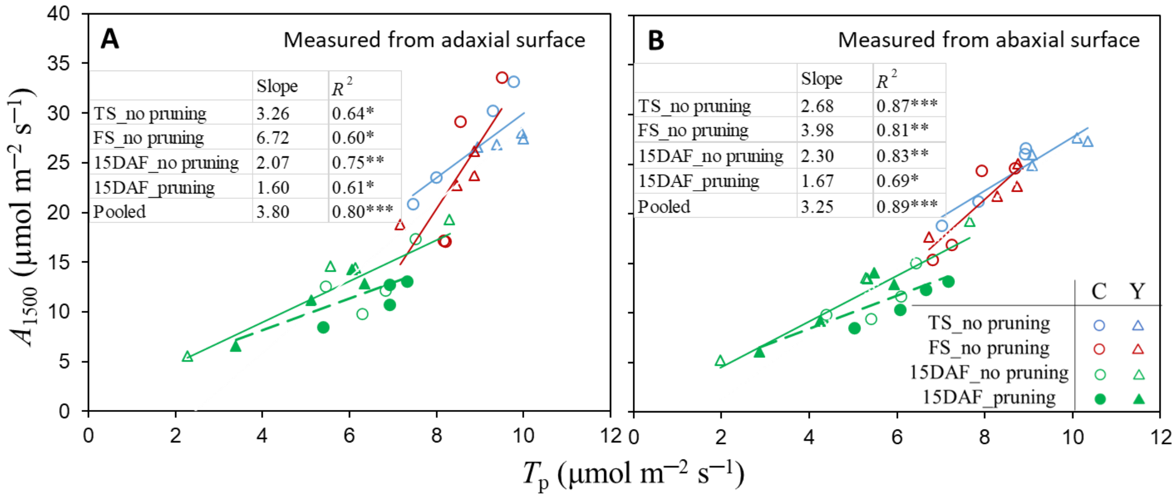

Fig. S5. Relationship between light-saturated leaf photosynthesis rate (A_{1500}) and the rate of triose phosphate utilisation (T_p) based on adaxial (A) and abaxial (B) measurements. Data represent the values for rice control (C) genotypes (circles) and yellower-leaf (Y) variant genotypes (triangles) of these intact (open symbols) and panicle-pruned (filled symbols) plants from tillering (TS, blue), flowering (FS, red), and 15 d after flowering (15DAF, green) stage in the 2022 experiment. Each point represents the mean of three or four replicates. Linear regressions were fitted for each stage and pooled data with the significance of correlation shown by asterisks: * $P < 0.05$, ** $P < 0.01$, *** $P < 0.001$.

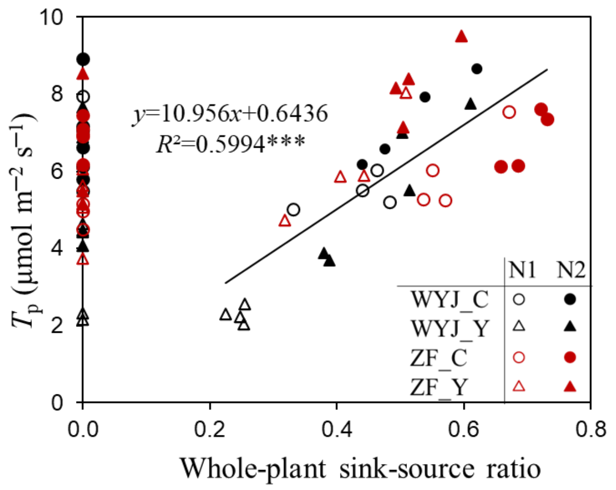

Fig. S6. Relationship between the rate of triose phosphate utilisation (T_p, based on measurements on the adaxial leaf surface) and whole-plant sink-source ratio. Here, following Fabre et al. (2020), the ratio of flag leaf area (source) to the fertile spikelet number of the panicle (sink) on the culm was used as an indicator of the whole-plant sink-source ratio (also see the text). Data are for rice control (C) genotypes (circles) and yellower-leaf (Y) variant genotypes (triangles) from grain-filling stage under low-nitrogen (N1, open symbols) and high-nitrogen (N2, filled symbols) levels in the 2022 experiment, with cv. Wuyunjing 3 (WYJ) in black and cv. Zhefu 802 (ZF) in red. For those plants with panicle pruning, we define their sink-source ratio to be zero, so all their data points fall on the Y axis. Linear regression was fitted for data (representing no pruning) with the significance of correlation shown by asterisks: *** $P < 0.001$.

Table S1. Modelled photosynthetic parameters (*δ* – the ratio of carboxylation resistance to mesophyll resistance; T_p – the rate of TPU; α_S – the proportion of glycolate carbon exported from photorespiratory pathway in the form of serine) for rice control (C) genotypes and their yellower-leaf (Y) variant genotypes at three stages under low-nitrogen (N1) and high-nitrogen (N2) levels measured from both sides of the leaves in the 2022 experiment.

Stage	Pruning level	N level	Background	Genotype	Adaxial surface			Abaxial surface		
					δ	T_p	α_S	*δ*	T_p	α_S
						μmol m^{-2} s^{-1}	-		μmol m^{-2} s^{-1}	-
Tillering	No pruning	N1	WYJ	C	1.13 ± 0.12	9.28 ± 0.49	0.241 ± 0.023	0.95 ± 0.15	8.90 ± 0.19	0.180 ± 0.019
			WYJ	Y	1.49 ± 0.28	10.00 ± 0.14	0.109 ± 0.032	1.07 ± 0.11	10.10 ± 0.51	0.107 ± 0.018
			ZF	C	1.16 ± 0.18	7.46 ± 0.36	0.284 ± 0.032	1.26 ± 0.19	7.02 ± 0.17	0.242 ± 0.039
			ZF	Y	1.22 ± 0.17	9.39 ± 0.39	0.108 ± 0.010	1.10 ± 0.15	9.09 ± 0.44	0.097 ± 0.033
		N2	WYJ	C	0.95 ± 0.10	9.76 ± 0.25	0.248 ± 0.024	1.31 ± 0.14	8.93 ± 0.22	0.193 ± 0.018
			WYJ	Y	1.74 ± 0.17	9.96 ± 0.45	0.063 ± 0.025	1.37 ± 0.09	10.35 ± 0.24	0.073 ± 0.027
			ZF	C	1.15 ± 0.18	7.99 ± 0.12	0.371 ± 0.019	1.14 ± 0.09	7.85 ± 0.24	0.290 ± 0.027
			ZF	Y	1.28 ± 0.15	8.96 ± 0.41	0.206 ± 0.014	1.20 ± 0.15	9.08 ± 0.44	0.166 ± 0.016
Flowering	No pruning	N1	WYJ	C	0.73 ± 0.05	8.54 ± 0.28	0.287 ± 0.009	0.73 ± 0.09	7.92 ± 0.14	0.185 ± 0.018
			WYJ	Y	1.28 ± 0.14	7.17 ± 0.35	0.073 ± 0.015	1.90 ± 0.25	6.73 ± 0.22	0.064 ± 0.008
			ZF	C	0.72 ± 0.07	8.16 ± 0.19	0.242 ± 0.035	1.01 ± 0.07	6.80 ± 0.27	0.138 ± 0.020
			ZF	Y	1.00 ± 0.12	8.87 ± 0.42	0.152 ± 0.020	1.00 ± 0.08	8.74 ± 0.33	0.176 ± 0.028
		N2	WYJ	C	0.72 ± 0.04	9.50 ± 0.23	0.344 ± 0.022	0.86 ± 0.04	8.69 ± 0.23	0.180 ± 0.045
			WYJ	Y	1.59 ± 0.18	8.48 ± 0.48	0.103 ± 0.026	1.67 ± 0.12	8.29 ± 0.23	0.105 ± 0.041
			ZF	C	0.63 ± 0.04	8.20 ± 0.19	0.254 ± 0.013	0.74 ± 0.09	7.24 ± 0.27	0.169 ± 0.026
			ZF	Y	0.97 ± 0.11	8.88 ± 0.14	0.199 ± 0.021	1.11 ± 0.08	8.77 ± 0.28	0.156 ± 0.017
15 d after flowering	No pruning	N1	WYJ	C	1.11 ± 0.15	5.45 ± 0.22	0.170 ± 0.023	1.06 ± 0.11	4.37 ± 0.36	0.153 ± 0.018
			WYJ	Y	1.08 ± 0.09	2.28 ± 0.11	0.091 ± 0.012	1.13 ± 0.13	1.98 ± 0.46	0.094 ± 0.035
			ZF	C	1.26 ± 0.11	6.29 ± 0.58	0.018 ± 0.015	1.16 ± 0.14	5.40 ± 0.38	0.011 ± 0.009
			ZF	Y	0.95 ± 0.22	6.14 ± 0.69	0.119 ± 0.010	0.98 ± 0.15	5.35 ± 0.48	0.112 ± 0.046
		N2	WYJ	C	0.72 ± 0.12	7.51 ± 0.48	0.205 ± 0.026	1.17 ± 0.13	6.42 ± 0.42	0.181 ± 0.026
			WYJ	Y	1.26 ± 0.20	5.57 ± 0.81	0.156 ± 0.011	0.77 ± 0.10	5.31 ± 0.82	0.138 ± 0.033
			ZF	C	1.02 ± 0.04	6.82 ± 0.39	0.101 ± 0.019	1.90 ± 0.25	6.09 ± 0.16	0.093 ± 0.023
			ZF	Y	1.15 ± 0.22	8.31 ± 0.49	0.187 ± 0.018	1.07 ± 0.18	7.66 ± 0.47	0.156 ± 0.016
	Pruning	N1	WYJ	C	0.53 ± 0.06*	6.92 ± 0.51*	0.105 ± 0.006*	0.92 ± 0.10	6.66 ± 0.21*	0.048 ± 0.021*
			WYJ	Y	0.55 ± 0.21*	3.40 ± 0.67	0.084 ± 0.014	0.46 ± 0.22*	2.87 ± 0.88	0.085 ± 0.014
			ZF	C	1.18 ± 0.22	5.39 ± 0.53	0.015 ± 0.009	1.50 ± 0.19	5.03 ± 0.41	0.000 ± 0.000
			ZF	Y	0.96 ± 0.12	5.12 ± 0.51	0.075 ± 0.010*	0.83 ± 0.08	4.26 ± 0.37	0.017 ± 0.017
		N2	WYJ	C	0.78 ± 0.22	7.33 ± 0.71	0.077 ± 0.007*	1.40 ± 0.28	7.16 ± 0.45	0.062 ± 0.008*
			WYJ	Y	0.92 ± 0.19	6.06 ± 0.75	0.109 ± 0.007*	1.27 ± 0.26	5.48 ± 0.74	0.093 ± 0.013
			ZF	C	0.82 ± 0.07	6.92 ± 0.27	0.000 ± 0.000*	1.10 ± 0.12*	6.07 ± 0.16	0.000 ± 0.000*
			ZF	Y	0.91 ± 0.23	6.35 ± 0.76	0.015 ± 0.009*	0.95 ± 0.15	5.94 ± 0.70	0.006 ± 0.006*

The asterisks (*) represent significant differences ($P < 0.05$) for a given genotype-nitrogen combination between pruned and un-pruned plants at 15 d after flowering.

Table S2. Leaf photosynthetic characteristics (mean ± standard error of four replicates) for rice control (C) genotypes and their yellower-leaf (Y) variant genotypes at three stages under low-nitrogen (N1) and high-nitrogen (N2) levels measured from both sides of leaves in the 2022 experiment.

Stage	Pruning level	N level	Background	Genotype	Adaxial surface			Abaxial surface				
					SPAD	A_{1500} (μmol m^{-2} s^{-1})	PNUE (μmol g^{-1} N s^{-1})	SPAD	A_{1500} (μmol m^{-2} s^{-1})	PNUE (μmol g^{-1} N s^{-1})	SLA (m^2 kg^{-1})	SLN (g N m^{-2})
Tillering	No pruning	N1	WYJ	C	42.9 ± 0.7	30.3 ± 1.6	25.8 ± 0.8	42.9 ± 0.5	26.1 ± 0.6	22.5 ± 1.2	24.9 ± 0.4	1.48 ± 0.06
			WYJ	Y	18.1 ± 1.1	27.4 ± 0.4	24.3 ± 0.7	18.5 ± 1.3	27.6 ± 1.3	24.4 ± 0.6	33.4 ± 1.9	1.43 ± 0.03
			ZF	C	30.9 ± 0.9	20.9 ± 1.6	24.3 ± 1.1	30.4 ± 0.9	18.8 ± 0.3	22.2 ± 1.1	27.3 ± 0.4	1.16 ± 0.04
			ZF	Y	21.2 ± 1.0	26.8 ± 1.5	27.2 ± 1.0	21.2 ± 0.9	24.8 ± 1.3	25.3 ± 1.1	29.6 ± 0.3	1.28 ± 0.04
		N2	WYJ	C	43.9 ± 0.9	33.2 ± 0.9	26.2 ± 0.7	43.2 ± 0.9	26.6 ± 0.5	21.1 ± 0.8	26.7 ± 1.4	1.57 ± 0.06
			WYJ	Y	18.2 ± 0.8	28.0 ± 1.3	25.2 ± 0.5	17.6 ± 1.0	27.3 ± 0.7	24.7 ± 1.6	33.8 ± 0.9	1.41 ± 0.07
			ZF	C	33.8 ± 1.1	23.6 ± 1.0	25.5 ± 0.5	33.4 ± 0.9	21.3 ± 0.6	23.3 ± 1.0	27.7 ± 0.2	1.23 ± 0.04
			ZF	Y	21.1 ± 0.6	26.5 ± 1.5	29.3 ± 0.7	21.0 ± 0.3	25.9 ± 0.6	28.8 ± 1.4	30.7 ± 1.6	1.21 ± 0.05
Flowering	No pruning	N1	WYJ	C	45.7 ± 0.7	29.1 ± 1.2	21.6 ± 1.0	45.5 ± 0.8	24.4 ± 1.7	18.0 ± 1.1	21.3 ± 0.5	1.65 ± 0.04
			WYJ	Y	12.6 ± 0.6	18.7 ± 1.5	15.9 ± 1.8	12.7 ± 0.5	17.6 ± 0.5	14.9 ± 1.0	25.4 ± 0.9	1.50 ± 0.08
			ZF	C	33.9 ± 0.1	17.2 ± 0.6	20.0 ± 1.0	33.8 ± 0.2	15.4 ± 0.7	17.8 ± 0.2	21.0 ± 0.3	1.17 ± 0.04
			ZF	Y	16.7 ± 0.7	23.7 ± 1.5	23.0 ± 0.4	16.6 ± 1.0	22.7 ± 0.6	22.4 ± 1.9	22.4 ± 0.2	1.33 ± 0.08
		N2	WYJ	C	49.7 ± 0.6	33.6 ± 0.7	21.0 ± 0.5	49.6 ± 0.7	24.6 ± 0.5	15.4 ± 0.5	20.3 ± 0.2	1.91 ± 0.06
			WYJ	Y	14.9 ± 0.8	22.7 ± 1.9	17.2 ± 1.8	15.2 ± 0.9	21.8 ± 1.5	16.3 ± 1.2	26.0 ± 1.1	1.64 ± 0.08
			ZF	C	35.9 ± 0.3	17.1 ± 0.8	17.7 ± 0.7	35.7 ± 0.3	16.9 ± 0.6	17.5 ± 0.5	21.7 ± 0.6	1.27 ± 0.02
			ZF	Y	20.2 ± 1.4	26.1 ± 1.4	23.2 ± 0.7	19.9 ± 1.5	25.0 ± 0.7	23.1 ± 1.8	23.3 ± 0.5	1.40 ± 0.06
15 d after flowering	No pruning	N1	WYJ	C	42.2 ± 1.0	12.6 ± 0.8	17.2 ± 1.3	41.8 ± 1.0	9.9 ± 1.3	13.8 ± 2.3	21.9 ± 0.9	1.04 ± 0.06
			WYJ	Y	4.6 ± 1.3	5.5 ± 0.6	5.6 ± 0.4	4.4 ± 1.2	5.2 ± 1.3	5.3 ± 1.2	26.8 ± 0.3	1.28 ± 0.05
			ZF	C	29.3 ± 1.0	9.9 ± 0.9	17.5 ± 0.9	30.0 ± 0.9	9.4 ± 1.1	16.6 ± 1.2	21.5 ± 0.4	0.87 ± 0.07
			ZF	Y	11.2 ± 1.4	14.4 ± 1.7	19.5 ± 0.9	10.7 ± 1.4	13.4 ± 1.7	18.2 ± 1.4	23.5 ± 0.4	1.03 ± 0.05
		N2	WYJ	C	45.5 ± 0.9	17.4 ± 1.9	19.1 ± 1.3	45.2 ± 0.7	15.1 ± 1.5	16.9 ± 1.8	21.5 ± 0.5	1.21 ± 0.06
			WYJ	Y	10.2 ± 1.9	14.5 ± 2.2	13.2 ± 1.5	10.7 ± 1.8	13.5 ± 2.4	12.3 ± 1.7	25.9 ± 0.9	1.38 ± 0.07
			ZF	C	34.2 ± 0.5	12.1 ± 1.1	17.4 ± 0.7	34.3 ± 0.4	11.7 ± 0.8	16.8 ± 0.6	21.7 ± 0.7	0.99 ± 0.04
			ZF	Y	17.9 ± 1.2	19.3 ± 1.3	21.0 ± 0.8	18.0 ± 1.5	19.2 ± 1.9	20.8 ± 1.3	23.8 ± 0.2	1.22 ± 0.03
	Pruning	N1	WYJ	C	45.3 ± 1.5	12.7 ± 1.3	12.0 ± 1.1*	45.4 ± 1.5	12.4 ± 1.0	11.9 ± 1.4	18.2 ± 0.8*	1.36 ± 0.07*
			WYJ	Y	8.1 ± 2.4	6.5 ± 1.8	4.9 ± 1.1	7.0 ± 2.5	6.0 ± 2.4	4.4 ± 1.5	22.8 ± 0.8*	1.57 ± 0.09*
			ZF	C	31.2 ± 0.9	8.5 ± 1.0	12.1 ± 0.3*	30.8 ± 0.9	8.5 ± 0.5	12.3 ± 0.8*	18.8 ± 0.4*	1.00 ± 0.08
			ZF	Y	12.0 ± 1.0	11.1 ± 0.9	9.7 ± 0.8*	11.9 ± 0.7	9.2 ± 0.6	8.0 ± 0.6*	19.0 ± 0.8*	1.45 ± 0.08*
		N2	WYJ	C	48.6 ± 0.3*	13.1 ± 1.9	10.6 ± 1.2*	49.1 ± 0.5*	13.2 ± 1.3	10.8 ± 0.9*	18.4 ± 0.5*	1.52 ± 0.07*
			WYJ	Y	10.3 ± 2.2	14.2 ± 2.1	10.0 ± 1.3	10.7 ± 2.0	14.0 ± 2.6	9.9 ± 1.7	23.5 ± 0.3*	1.71 ± 0.02*
			ZF	C	34.4 ± 0.8	10.7 ± 0.7	11.9 ± 0.4*	33.8 ± 0.9	10.3 ± 0.9	11.5 ± 0.7*	18.3 ± 0.7*	1.20 ± 0.06*
			ZF	Y	13.0 ± 1.5*	12.8 ± 1.7*	9.3 ± 0.7*	12.5 ± 1.3*	12.8 ± 1.2*	9.4 ± 0.5*	19.4 ± 0.4*	1.66 ± 0.08*

A_{1500}, photosynthetic rate at saturated light of 1500 μmol m^{-2} s^{-1} and ambient CO_2 level; PNUE, leaf photosynthetic nitrogen-use efficiency, defined as: $\text{PNUE} = \frac{A_{1500}}{\text{SLN} - n_b}$, where SLN is specific leaf nitrogen, and n_b represents a base leaf nitrogen content (a value of 0.23 g N m^{-2} for all the rice genotypes from Zhou et al., 2023); SLA, specific leaf area. The asterisks (*) represent significant differences ($P < 0.05$) for a given genotype-nitrogen combination between pruned and un-pruned plants at 15 d after flowering.

Table S3. Summary of analysis of variance of leaf photosynthetic variables: the rate of TPU (T_p), the proportion of glycolate carbon exported from photorespiratory pathway in the form of serine (α_S), the ratio of carboxylation resistance to mesophyll resistance (δ), photosynthetic rate at saturated light of 1500 μmol m^{-2} s^{-1} and ambient CO_2 level (A_{1500}), leaf photosynthetic nitrogen-use efficiency (PNUE), chlorophyll content indicator (SPAD), specific leaf nitrogen (SLN), and specific leaf area (SLA), in response to genotype, panicle pruning, abaxial vs adaxial measurements, N level, three-stages' measurements, and their interactions (if applicable). Data are from the 2022 experiment, with values of these parameters shown in Tables S1-S2.

Variable	T_p	α_S	δ	A_{1500}	PNUE	SPAD	SLN	SLA
Genotype	***	***	***	***	***	***	***	***
Pruning	ns	***	**	***	***	ns	***	***
Adaxial vs Abaxial	***	***	*	***	***	ns	-	-
N level	***	***	ns	***	***	***	***	ns
Stage	***	ns	***	***	***	ns	***	***
Genotype × Pruning	***	***	ns	**	***	***	*	ns
Genotype × Adaxial vs Abaxial	ns	***	*	**	*	ns	-	-
Genotype × N level	***	ns	ns	**	***	ns	ns	ns
Genotype × Stage	***	***	***	***	**	***	ns	*
Pruning × Adaxial vs Abaxial	ns	ns	ns	ns	ns	ns	-	-
Pruning × N level	ns	***	ns	ns	*	**	ns	ns
Adaxial vs Abaxial × N level	ns	ns	ns	ns	ns	ns	-	-
Adaxial vs Abaxial × Stage	ns	ns	*	ns	ns	ns	-	-
N level × Stage	ns	ns	ns	ns	ns	**	*	ns

In the analysis of variance, the significance is shown by asterisks: ns – no significance, * $P < 0.05$, ** $P < 0.01$, *** $P < 0.001$, according to the LSD test. Note, we use "genotype" rather than "yellower-leaf modification" as a fixed factor mainly because yellower-leaf modification produces different effects on leaf photosynthetic physiology between WYJ and ZF backgrounds (see Results).

References

Busch FA. 2020. Photorespiration in the context of Rubisco biochemistry, CO_2 diffusion and metabolism. *The Plant Journal* **101**: 919−939.

Busch FA, Sage RF, Farquhar GD. 2018. Plants increase CO_2 uptake by assimilating nitrogen via the photorespiratory pathway. *Nature Plants* **4**: 46−54.

Fabre D, Dingkuhn M, Yin X, Clément-Vidal A, Roques S, Soutiras A, Luquet D. 2020. Genotypic variation in source and sink traits affects the response of photosynthesis and growth to elevated atmospheric CO_2. *Plant, Cell & Environment* **43**: 579−593.

Yin X, Busch FA, Struik PC, Sharkey TD. 2021. Evolution of a biochemical model of steady-state photosynthesis. *Plant, Cell & Environment* **44**: 2811−2837.

Zhou Z, Struik PC, Gu J, van der Putten PEL, Wang Z, Yin X, Yang J. 2023. Enhancing leaf photosynthesis from altered chlorophyll content requires optimal partitioning of nitrogen. *Crop and Environment* **2**: 24-36 (DOI: 10.1016/j.crope.2023.02.001).
